# Cognitive assessment methods and outcomes following shunt surgery in idiopathic normal pressure hydrocephalus (iNPH): a systematic review and meta-analysis

**DOI:** 10.1186/s12987-026-00808-3

**Published:** 2026-05-11

**Authors:** Lisa M. Healy, Jeffrey Tooze, David Quist, Priya Varma, Christopher Carswell, Rocío Fernández-Méndez, John D. Pickard, Peter Smielewski, Alexis J. Joannides

**Affiliations:** 1 Division of Neuroscience, Addenbrooke’s Hospital, Cambridge, CB2 0QQ UK; 2 NIHR HealthTech Research Centre in Brain Injury, Cambridge, UK; 3 Department of Clinical Neurosciences, University of Cambridge, Cambridge, UK; 4 Imperial College Healthcare NHS Trust, London, UK; 5 Faculty of Health Sciences, Universitat Jaume I, Castelló de la Plana, Spain

**Keywords:** Idiopathic normal pressure hydrocephalus, Shunt surgery, Cognitive outcomes, Cognitive tests

## Abstract

**Introduction:**

Core cognitive deficits in iNPH include slowed information processing, psychomotor slowing and executive dysfunction. However, cognitive outcomes following shunt surgery are not well understood. This review synthesised evidence on cognitive assessment methods and outcomes following shunt surgery in iNPH.

**Methods:**

PubMed, Scopus, PsycINFO and Web of Science were searched for peer-reviewed studies including adults with iNPH who underwent shunt surgery and had within-subject cognitive evaluations pre- and post-operatively. Key data were extracted and study quality was assessed. Random-effects meta-analyses were performed on pooled baseline and post-shunt difference scores for frequently reported cognitive tests with comparable outcome data.

**Results:**

Of 1,876 records, 195 met the inclusion criteria, comprising 11,445 patients. Cognitive evaluation methods ranged from subjective reports and NPH grading scales to brief screening tools and comprehensive test batteries. Over 193 distinct tests were reported and 54.4% of studies did not formally assess any core iNPH cognitive deficits. Post-shunt improvement rates, follow-up times and criteria for defining improvement varied widely. Eighty-five studies contributed data to meta-analyses of ten outcomes. Pooled estimates indicated post-shunt cognitive improvement, with Trail Making Test-A, Grooved Pegboard-Dominant and Trail Making Test-B showing changes exceeding thresholds for clinically significant improvement.

**Conclusions:**

Cognitive assessment in iNPH is highly heterogeneous and frequently omits core domains, limiting detection of treatment effects. When domain-relevant cognitive measures are used, shunt surgery is associated with statistically and clinically significant cognitive improvement. These findings highlight the need for standardised iNPH-appropriate cognitive evaluation tools with validated criteria for detecting clinically meaningful change and have direct implications for clinical assessment, interpretation of shunt response and the selection of cognitive endpoints in future interventional studies.

**Supplementary Information:**

The online version contains supplementary material available at 10.1186/s12987-026-00808-3.

## Summary box

What is already known on this topic

What this study adds

How this study might affect research, practice or policy

Cognitive outcomes after shunt surgery for idiopathic normal pressure hydrocephalus (iNPH) have been inconsistently reported, with cognitive improvement reported less reliably than gait outcomes, in the context of highly variable assessment practices across centres.

This systematic review of 195 studies (11,445 patients) shows substantial heterogeneity in iNPH cognitive assessment and demonstrates that when tests sensitive to frontal–subcortical dysfunction are used, shunt surgery is associated with statistically and clinically meaningful cognitive improvement. Widely used dementia screening tools, including the MMSE and MoCA, show changes largely within expected practice-effect ranges and do not adequately capture core iNPH cognitive deficits.

These findings demonstrate the need to standardise cognitive assessment in iNPH using iNPH-appropriate tools with validated metrics for determining clinically meaningful improvement. This will enable robust trial endpoints and accurate evaluation of cognitive benefits of shunting in routine clinical practice.

**Supplementary Information:**

The online version contains supplementary material available at 10.1186/s12987-026-00808-3.

## Introduction

Idiopathic normal pressure hydrocephalus (iNPH) primarily affects older adults and is characterised by gait and balance disturbance, cognitive impairment and urinary incontinence in the context of ventriculomegaly [1, 2]. The pattern of cognitive impairment typically reflects frontal-subcortical dysfunction, with core deficits in information processing speed, psychomotor speed, executive functioning and apathy, which can impact daily functioning and quality of life [3–7].

iNPH is often underdiagnosed and misdiagnosed due to its overlapping symptoms with neurodegenerative conditions and because gait, cognitive and bladder symptoms are common in older adults and may be mistaken for normal ageing [8–10]. Surgical CSF diversion by shunt insertion can alleviate iNPH symptoms [11, 12]. Whilst gait changes have been widely documented, the effect of shunt surgery on cognitive symptoms is not well understood [13–15], and cognitive impairment has been cited as the least likely symptom to improve post-surgery [16]. This uncertainty may reflect the high prevalence of comorbid neurodegenerative and cerebrovascular pathologies in iNPH patients, which can independently contribute to cognitive decline and attenuate measurable cognitive gains post-shunt [17–19]. Accurate cognitive assessment may therefore be important for diagnostic accuracy and for detecting treatment response [14].

Currently, there is no internationally standardised approach to cognitive assessment in iNPH for clinical or research purposes, leading to variability in the tests administered across centres [20, 21]. On this background, the purpose of the current review was to provide a comprehensive and up-to-date synthesis of the literature on cognitive outcomes in iNPH. Specifically, the aims were to understand (i) how cognition is assessed pre-post shunt, (ii) the cognitive outcomes following shunt surgery, and (iii) the methods for defining post-shunt cognitive improvement. Meta-analyses were conducted on the most frequently used tests with extractable cognitive data.

## Methods

### Search strategy

PubMed, Scopus, PsycINFO and Web of Science were searched from inception to January 2021, with the search subsequently updated to 25 September 2025. The following terms were used, adapted for each database as appropriate: (iNPH OR idiopathic normal pressure hydrocephalus OR NPH OR normal pressure hydrocephalus) AND (shunt* OR CSF diversion OR surgery) AND (cognition OR cognitive OR neuropsychological assessment OR neuropsychological outcome OR shunt outcome). Reference lists of the included studies were manually screened for additional eligible studies. The protocol was registered with PROSPERO (ID CRD42021296112). The review followed the Preferred Reporting Items for Systematic Reviews and Meta-Analyses (PRISMA) guidelines [22].

### Study selection

Two reviewers [LH, DQ] independently screened abstracts to identify articles to retrieve in full. Discrepancies were resolved by discussion involving a third reviewer [PV]. The inclusion criteria were: (1) peer-reviewed studies in English, (2) adults who underwent shunt surgery for suspected iNPH, (3) within-subject evaluations of cognition before and after shunt surgery, (4) specified cognitive evaluation method, and (5) extractable cognitive outcomes post-surgery. Studies including multiple diagnoses were required to report independently extractable iNPH data and studies reporting multiple surgical procedures were required to provide shunt-specific outcomes. Exclusion criteria included: (1) case studies, (2) patients with secondary NPH or cases where the exact nature of hydrocephalus was unclear, (3) composite improvement scores in the iNPH symptom triad with no extractable cognitive data, (4) conference proceedings, and (5) cohorts treated exclusively with endoscopic third ventriculostomy (ETV).

### Data extraction

Data extraction was completed and validated by two reviewers [LH, JT]. Key descriptive data were extracted: first author, publication year, country, study design, follow-up timeframe, cognitive evaluation method, improvement criteria, sample size and sample characteristics (mean/median age, sex and symptom duration). For RCTs and designs involving multiple conditions or healthy controls, only pre-post shunt cognitive data from iNPH participants was extracted. Demographic and cognitive outcome data were not duplicated when the same patient cohort was reported across multiple publications.

### Quality assessment

Methodological quality was assessed using the Methodological Index for Non-Randomised Studies (MINORS) [23]. Studies were evaluated across eight domains: study aim, selection bias (consecutive patients), prospective design, appropriate data collection methods, unbiased assessment (blinding), adequacy of follow-up, attrition and prospective sample size calculation, each rated as weak (0), moderate (1), or strong (2). In line with the focus of the present review, domains relating to study aims and outcome assessment were interpreted with reference to the assessment of cognitive outcomes (Supplementary Table 1).

### Meta-analyses

Meta-analyses were conducted on the pooled baseline and difference scores for the following tests: Digit Span Backward (DSB), Digit Span Forward (DSF), Frontal Assessment Battery (FAB), Grooved Pegboard Test-Dominant hand (GPB-D), Mini-Mental State Examination (MMSE), Montreal Cognitive Assessment (MoCA), Rey Auditory Verbal Learning Test (RAVLT), Trail Making Test-A (TMT-A) and Trail Making Test-B (TMT-B). These tests were selected based on having at least five studies with extractable mean (SD) or median (IQR, range) data from comparable test versions. Separate outcome data were available for the RAVLT Learning and Delayed Recall subtests, which were treated as distinct cognitive outcome measures for meta-analysis purposes. Insufficient comparable data were available for the Stroop and Letter Fluency tests due to heterogeneity in test versions, languages, scoring procedures or incomplete reporting of relevant summary statistics [24]. For studies with multiple follow-ups, data from the last available post-shunt assessment were used. Data from multiple publications were not duplicated. Analyses were performed using IBM SPSS v30.

Where reported, medians (IQR, range) were converted to means (SD) using the Wan method [25], with Box-Cox transformations applied to skewed data [26]. For case-series studies reporting individual pre- and post-shunt scores, group means and SDs were calculated manually from the raw data provided.

To account for variability across studies, random-effects meta-analyses were performed using the Restricted Maximum Likelihood (REML) method [27]. Pooled difference scores were used as outcomes to account for sample sizes and variances. Effect sizes were expressed as unstandardised mean differences. Heterogeneity was assessed using the I² statistic [28]. Publication bias was examined using funnel plots and Egger’s regression test [29]. Trim-and-fill was applied where indicated, to estimate the impact of potentially missing studies [30]. Results are presented as pooled difference scores with 95% confidence intervals.

#### Clinical significance

Clinically significant improvement was defined a priori using two approaches: (i) age-matched, distribution-based thresholds derived from pooled normative standard deviations (0.3 SD = small, 0.5 SD = moderate, 1.0 SD = large clinical change), and (ii) age-corrected z-score change relative to normative data (≥ 1 z-score). These criteria allow statistically significant pooled effects to be interpreted against benchmarks of clinically meaningful cognitive improvement [31–35].

#### Sensitivity analyses

Sensitivity analyses were conducted to assess the robustness of pooled estimates and the influence of outlier or high-variance studies. Stepwise exclusions were performed for each cognitive outcome, prioritising studies with extreme mean differences, large standard errors, or visually identified influence in forest or funnel plots. Outlier studies were also identified based on conceptual and psychometric criteria, such as marked directional inconsistency with other outcomes within the same study, or extreme variance shifts. After each exclusion, random-effects models were re-estimated as described above. Sensitivity analyses were considered confirmatory when the direction and significance of pooled effects were preserved [24, 36].

**Fig. 1:**
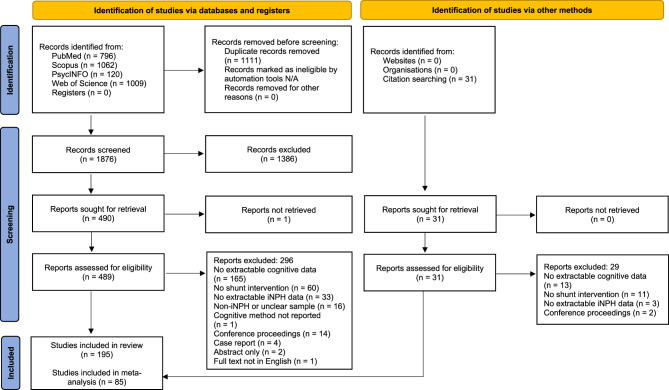
PRISMA diagram

## Results

### Search identification

After removing duplicates, 1,876 unique records were identified from the systematic search (Fig. 1). Titles and abstracts were screened and 1,386 records were excluded. One article was not accessible for full-text review and could not be retrieved from the corresponding author. Full texts of the remaining 489 studies were assessed for eligibility. An additional 31 studies were identified from backward citation searching. In total, 195 studies met the inclusion criteria and were included in the final review. A full reference list is provided in the Supplementary Material. Study characteristics and participant demographics are summarised in Table 1.

### Quality assessment (MINORS)

**Fig. 2:**
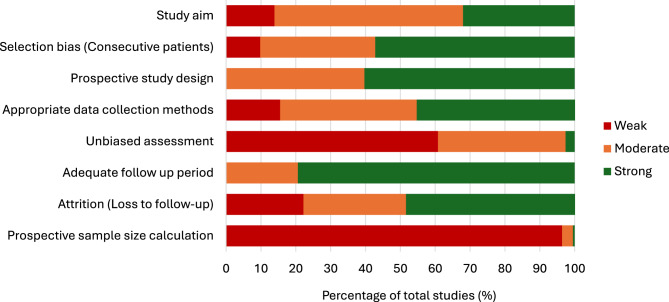
Distribution of MINORS quality assessment ratings across methodological domains in the 195 included studies

Application of the MINORS criteria demonstrated heterogeneous methodological quality across domains (Fig. 2; Supplementary Table 1). Weak ratings were most frequently observed for unbiased outcome assessment (blinding) and prospective sample size calculation. Prospective design, consecutive patient inclusion and adequacy of follow-up were more commonly rated as moderate to strong.

**Table 1:** Study and participant characteristics

Characteristic	Summary
Total studies (n)	195
Study design, n (%)	
Prospective	118 (60.5%)
Retrospective	77 (39.5%)
Participants (n)	11,445
Sex, n (%)	
Male	6,459 (56.4%)
Female	4,555 (39.8%)
Unspecified	431 (3.8%)
Median age (years)	70–77
Symptom duration at baseline	
Median	24 months
Range	1–72 months
Countries represented (*n* = 26)	Argentina, Australia, Austria, Belgium, Brazil, Canada, China, Czechia, Denmark, Finland, Germany, Greece, Hungary, Italy, Japan, Netherlands, Norway, Portugal, Republic of Korea, Spain, Sweden, Switzerland, Taiwan, Turkey, UK, USA

Note. Median age and symptom duration were available for subsets of studies

### Cognitive evaluation before and after shunt surgery

#### Timing of testing

**Fig. 3:**
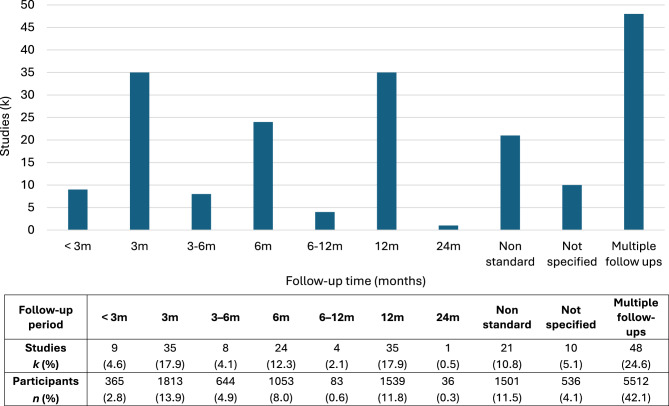
Distribution of follow-up times in 195 studies with post-shunt cognitive data

Of the 195 included studies, 137 (70.3%) reported cognitive data from a single post-operative follow-up, 29 (14.9%) reported two follow-ups, 19 (9.7%) reported three or more follow-ups (up to a maximum of six) and 10 (5.1%) did not specify the follow-up time used. Modal follow-up times were 3 and 12 months (range 2 days to 8–10 years). The distribution of follow-up intervals is presented in Fig. 3.

#### Cognitive evaluation methods

Subjective ratings by patients, clinicians and/or carers were reported in 89 studies (45.6%). Subjective ratings were used as a standalone method in 30 studies (15.4%) and in combination with cognitive tests in 59 (30.3%) studies. Standardised symptom measures or structured interviews were primarily used with carers (13/20 studies).

61 studies (31.3%) had extractable cognitive data from NPH grading scales, including various iterations of the Japanese NPH Grading Scale–Cognition (JNPHGS-Cognition; 39/195, 20.0%), Hellstrom iNPH Grading Scale-Neuropsychology (12/195, 6.2%) and the Sahuquillo NPH Scale-Cognitive Function (10/195, 5.1%). These NPH grading scales were based on subjective clinician ratings, a composite score generated from cognitive tests, or a combination of subjective and objective test data. A further seven studies used author-specific grading scales.

165 studies (84.6%) used at least one cognitive testing method. Brief non-specific dementia screening tools (e.g. MMSE, MoCA) were used as standalone cognitive tests in 63 studies (32.3%). 102 studies (52.3%) used cognitive test batteries containing two or more tests. Test batteries were composed of a median of five tests (IQR 3–10, range 2–33). Two studies did not specify the number of tests used.

Table 2 lists the most frequently reported cognitive tests, with extractable cognitive outcome data. At least 193 distinct cognitive tests were identified across the included studies, 127 of which appeared in one study only. These tests assessed a broad range of cognitive functions spanning attention and working memory, executive function, language and verbal fluency, memory and orientation, information processing speed, psychomotor speed, reaction time, social cognition and visuospatial function. The reported cognitive tests are listed in full in Supplementary Table 2.

More than half of the included studies (106/195, 54.4%), did not formally test any of the core cognitive deficits observed in iNPH. 58 studies (29.7%) reported test batteries including both a measure of executive function and processing speed or psychomotor speed, which are core cognitive deficits observed in iNPH. Seven studies reported a measure of reaction time, a related indicator of the cognitive slowing characteristic of iNPH. The most common cognitive evaluation tool was the MMSE dementia screen, with extractable cognitive data in 128 studies (65.6%). Regarding test modality, most studies used conventional paper and pencil methods to administer cognitive tests. Nine studies (4.6%) reported a computerised assessment method.

**Table 2:** Most frequently used cognitive tests with extractable data

Test	Main cognitive domains	Studies		Participants	
		k	(%)	*n*	(%)
MMSE	Dementia screen	128	(65.6)	8227	(71.9)
TMT-A	Psychomotor / processing speed	38	(19.5)	1342	(11.7)
FAB	Executive function screen	35	(17.9)	969	(8.5)
RAVLT	Memory	32	(16.4)	1408	(12.3)
Stroop Interference	Executive function	32	(16.4)	1334	(11.7)
Grooved Pegboard Test	Psychomotor speed	27	(13.8)	1170	(10.2)
Digit Span Backward	Attention / working memory	26	(13.3)	993	(8.7)
Digit Span Forward	Attention	26	(13.3)	993	(8.7)
TMT-B	Executive function	25	(12.8)	971	(8.5)
Semantic Fluency	Verbal fluency / language	22	(11.3)	804	(7.0)
Stroop Colour Naming	Processing speed	22	(11.3)	1293	(11.3)
Letter Fluency	Verbal fluency / executive function	20	(10.3)	585	(5.1)

Note. Percentages refer to the proportion of total studies (*k* = 195) and participants (*n* = 11,445). FAB = Frontal Assessment Battery, MMSE = Mini-Mental State Examination, TMT = Trail Making Test, RAVLT = Rey Auditory Verbal Learning Test. Digit Span, Letter Fluency, RAVLT, Semantic Fluency, Stroop, TMT-A, and TMT-B include all test versions and languages

### Definition of post-shunt cognitive improvement

Post-shunt cognitive improvement (PCI) was defined using heterogeneous criteria across studies. PCI was operationalised by improvement rate in 56 studies (28.7%), statistically significant sample-level improvement in 92 studies (47.2%) and a combination of both approaches in 47 studies (24.1%), based on at least one post-operative follow-up. Of the 103 (52.8%) studies that defined PCI using an improvement rate approach, 42 exclusively used subjective ratings, 52 used objective testing, and nine incorporated both methods.

Subjective ratings of PCI were reported either as dichotomous outcomes (improvement, no improvement; *n* = 43) or stratified using graded improvement scales (range 3–4 points; *n* = 8) rated by clinicians/researchers. For the JNPHGS-Cognition, the most common improvement threshold was ≥ 1 point.

Considerable variability was observed in the operational definitions of PCI among studies reporting objective test data. Most studies specified diagnostic rules applied to individual cognitive tests or test batteries. The most common criterion was improvement by a specified number of points or reduction in completion time (36/58 studies). 17 studies applied two or more diagnostic rules to define PCI. A validated improvement metric was reported in eight studies (z-score, t-score or SD change). Table 3 outlines the diagnostic rules applied.

### Post-shunt cognitive outcomes

**Fig. 4:**
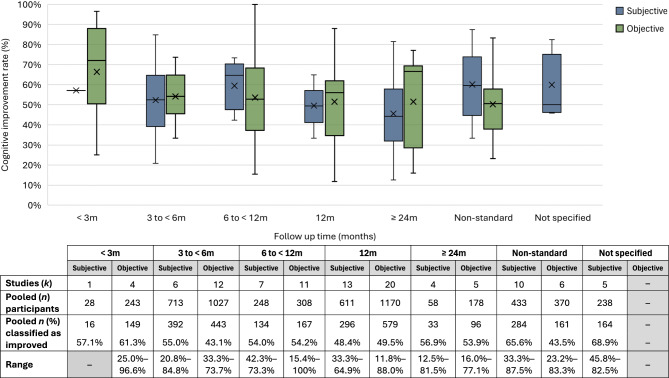
Pooled post-shunt cognitive improvement (PCI) rate in studies with extractable data. Box plots represent the median and interquartile range (25th–75th percentiles) for subjective and objective data at each follow-up period. Whiskers represent the range. The horizontal line within each box represents the median and the X indicates the mean value

Of the 139 studies defining PCI by statistical significance, 110 (79.1%) reported improvement in at least one cognitive parameter. Among studies reporting an improvement rate, the pooled PCI rate was 56.6% for subjective reports (range 12.5–87.5%) and 48.4% for cognitive testing (range 11.8–100%). Five studies also reported PCI rates based on combined subjective and objective data, yielding a pooled rate of 64.2% (range 39.5–85.5%). Pooled PCI rate at each follow-up period is presented in Fig. 4.

**Table 3:** Diagnostic rules used to define post-shunt cognitive improvement based on individual cognitive tests or test batteries in 58 studies

Individual cognitive tests	Studies (*n*)
Test improved by:	
Percentage (range 5–25%)	5
Specified points or completion time	36
≥ 1 SD	2
≥ 5 T-Score points	1
Test improved to a specific points threshold	1
Criteria NS	10

Batteries of cognitive tests	Studies *(n)*
≥ 1 SD improvement on ≥ 1/12 tests	1
≥ 1 SD improvement on ≥ 50% tests	3
≥ 25% improvement on ≥ 50% of tests	1
≥ 33.3% of tests in battery improved (Score NS)	1
≥ 50% of tests in battery improved (Score NS)	2
Composite score improved (Criteria NS)	6
Composite score improved by specified points	2
Composite score improved by ≥ 1 z-score	1
Executive function tests improved (Criteria NS)	1
Criteria NS	4

Note. 17 studies applied two or more diagnostic rules; SD = standard deviation; NS = not specified

### Meta-analyses of post-shunt cognitive change

Eighty-five studies met the inclusion criteria for the meta-analyses (Table 4). Extractable cognitive outcome data from ≥ 5 studies were available for DSB, DSF, FAB, GPB-D, MMSE, MoCA, RAVLT Learning, RAVLT Delayed Recall, TMT-A and TMT-B. Heterogeneity ranged from low to substantial (I² = 9.4–64.3%), with no outcome reaching the threshold for considerable heterogeneity. Egger’s test indicated publication bias for the MMSE (*p* = .029), with no evidence of bias for other measures. Trim-and-fill adjustment resulted in modest attenuation of pooled effects, which remained statistically significant.

All outcome measures showed pooled difference scores in the direction of cognitive improvement following shunt surgery. Statistical significance was therefore interpreted alongside predefined thresholds for clinically meaningful change. The magnitude of pooled effects varied across tests, with DSF (*p* = .129) the only outcome that did not reach statistical significance. Among the nine significant outcomes, mean difference scores for six measures (DSB, FAB, MMSE, MoCA, RAVLT Learning and RAVLT Delayed Recall) fell within ranges typically attributed to practice effects, test–retest variability or measurement error, rather than indicating genuine post-shunt cognitive change [37, 38].

**Fig. 5:**
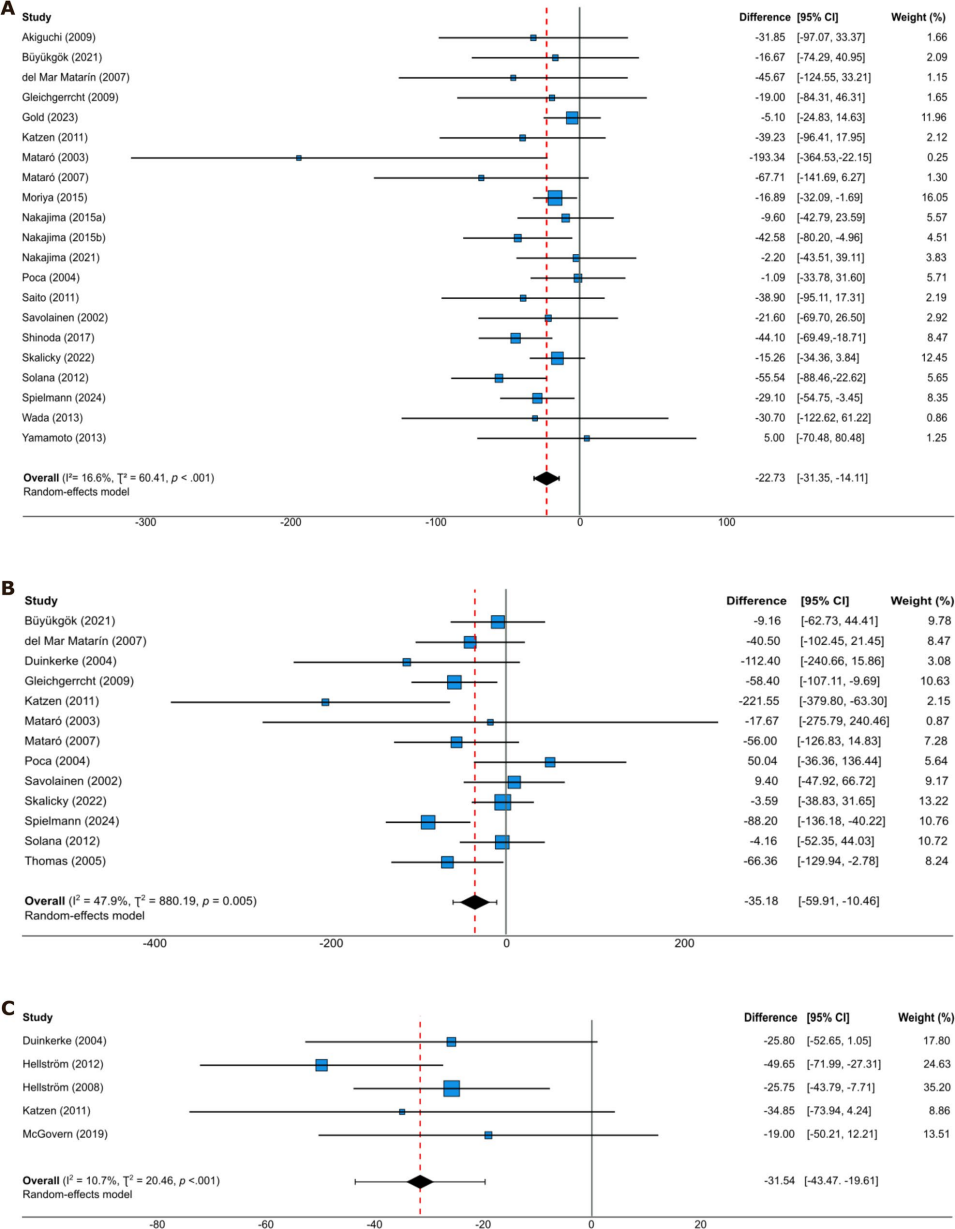
Forest plots of post-shunt difference scores for TMT-A, TMT-B and GPB-D. **A** Post shunt difference scores for TMT-A. **B** Post shunt difference scores for TMT-B. **C** Post shunt difference scores for GPB-D. Note. Pooled mean difference scores were calculated using a random-effects model. Mean difference estimates for individual studies are presented with corresponding 95% confidence intervals and percentage weights. For each test, the unit of measurement is completion time in seconds. Negative mean differences indicate improved performance (faster completion)

Three measures, TMT-A, GPB-D and TMT-B (for which faster completion time indicates better performance), demonstrated statistically and clinically significant improvements that exceeded all prespecified thresholds for clinically meaningful change [31–35]. Figure 5 presents the forest plots for TMT-A, GPB-D and TMT-B. Remaining outcomes are displayed in Supplementary Figs. 1–7.

Sensitivity analyses confirmed that pooled cognitive effects were robust across outcomes. Stepwise exclusion of influential or outlier studies and trim-and-fill analyses did not materially alter the direction or significance of any pooled estimates (Supplementary Table 3).

**Table 4:** Meta-analyses results for 85 studies

Cognitive outcome measure	Studies (k)	Estimated average pre-shunt score	Estimated average difference score	95% CI	*p*	Cochran’s Q (df; *p*)	I^2^ (%)	Between-study variance (τ²)	Egger’s test (*p*)	Clinically significant
**Executive function, attention and working memory**										
DSB	10	2.95	0.33	[0.10, 0.55]	0.005	26.24	64.3	0.07	0.781	No
		digits	digits			(9; 0.002)				
DSF	10	4.69	0.10	[-0.03, 0.24]	0.129	6.34	9.4	0.01	0.944	No
		digits	digits			(9; 0.705)				
FAB	24	11.18	1.56	[1.04, 2.08]	< 0.001	69.04	60.0	0.92	0.217	No
		points	points			(23; <0.001)				
TMT-B	13	234.44	-35.18	[-59.91, -10.46]	0.005	25	47.9	880.19	0.990	Yes
		seconds	seconds			(12; 0.015)				
**Processing speed / psychomotor speed**										
GPB-D	5	155.25	-31.54	[-43.47, -19.61]	< 0.001	3.74	10.7	20.46	0.272	Yes
		seconds	seconds			(4; 0.442)				
TMT-A	21	114.74	-22.73	[-31.35, -14.11]	< 0.001	22.05	16.6	60.41	0.252	Yes
		seconds	seconds			(20; 0.338)				
**Learning and memory**										
RAVLT Learning	12	23.70	5.58	[3.77, 7.39]	< 0.001	18.65	41.3	3.50	0.122	No
		words	words			(11; 0.068)				
RAVLT Delayed	11	2.75	1.37	[0.86, 1.88]	< 0.001	16.62	42.9	0.26	0.149	No
		words	words			(10; 0.083)				
**Global cognitive function**										
MoCA	7	19.06	1.75	[0.79, 2.71]	< 0.001	9.41	22.4	0.37	0.387	No
		points	points			(6; 0.152)				
MMSE	71	22.94	1.57	[1.23, 1.91]	< 0.001	149.95	61.0	0.95	0.029	No
		points	points			(70; <0.001)				
MMSE	88	–	1.07	[0.67, 1.47]	< 0.001	–	–	–	–	–
(imputed)			points							

## Discussion

This systematic review represents the largest pooled iNPH cohort to date evaluating cognitive outcomes, assessment methods and definitions of improvement following shunt surgery. Drawing on 195 studies, encompassing 11,445 patients from 26 countries, it combines subjective and objective assessment approaches to provide a comprehensive synthesis of cognitive change following shunt surgery. Critically, this review reveals that substantial methodological heterogeneity may have obscured true treatment effects. Importantly, when measures targeting core iNPH cognitive domains are employed, there is evidence of statistically and clinically significant cognitive improvement following shunt surgery.

### Heterogeneity in assessment and improvement criteria

Considerable heterogeneity was observed in both cognitive assessment methods and definitions of post-shunt cognitive improvement. Cognitive evaluation varied widely in test selection and timing of post-operative reassessment, with most studies (70.3%) reporting a single cognitive follow-up and few including longitudinal data. This is an important consideration given the chronic and progressive nature of iNPH, where cognitive outcomes may evolve over time [10].

Test battery composition was highly heterogeneous, with 127 of 193 reported tests used only once. More than half of studies failed to assess any core iNPH-related cognitive domains (processing speed, psychomotor speed and executive function), reflecting the absence of a standardised assessment approach. Commonly used dementia screening tools, such as the MMSE, were frequently used despite poor sensitivity to frontal–subcortical dysfunction and limited ability to detect change due to ceiling effects [39]. Furthermore, sensitive measures were embedded within global composite scores in several studies, potentially masking domain-specific improvement. This aligns with a review of four RCTs, which also reported a lack of assessment uniformity and noted that commonly used tools lack sensitivity to NPH-relevant cognitive change [13].

Heterogeneity in how cognitive improvement was defined further compounded these assessment issues. Many studies relied solely on statistical significance as evidence of improvement, which does not account for potential practice effects associated with repeated testing and may overlook clinically meaningful individual-level change. Adjustment for multiple comparisons was inconsistently reported in these analyses, representing an additional methodological limitation when interpreting sample-level findings. Few studies stratified analyses by baseline cognitive impairment, despite not all patients with iNPH presenting with cognitive deficits [2]. This may have diluted observable post-shunt effects among those with baseline cognitive deficits and obscured clinically meaningful change.

Arbitrary thresholds were commonly applied to define improvement rates. Only eight studies used recognised, norm-referenced approaches (z-score, SD or T-score change metrics), which allow change to be interpreted relative to expected variability in age-matched populations. These approaches provide a more objective and interpretable basis for estimating clinically meaningful change and can be applied at both the individual and sample level. In contrast, unstructured thresholds (or reliance on statistical significance alone) do not account for expected variability or measurement error and are therefore at risk of misclassifying normal variation or practice effects as improvement. No study reported use of reliable change indices, which incorporate test–retest reliability to distinguish true change from measurement error [34, 35]. Similar considerations apply when cognitive testing is repeated over short intervals, such as during CSF diagnostic procedures, where practice effects may increase the risk of false positive findings if appropriate thresholds for clinically meaningful change are not applied.

Subjective cognitive improvement was often poorly defined, with most studies relying on dichotomised outcomes and unstructured ratings, rendering them insensitive to subtle change and vulnerable to recall bias [40]. Although the JNPHGS-Cognition offers a structured grading system, its categories are not specific to iNPH and may be most useful when interpreted alongside targeted cognitive testing [41].

Together, this methodological and definitional heterogeneity contributes to variability in reported improvement rates, including wide variation within objectively assessed outcomes. This suggests that test selection and improvement criteria are key contributors to outcome heterogeneity, which could potentially obscure true treatment response and complicate interpretation of clinically meaningful change across studies.

However, heterogeneity in cognitive outcomes likely reflects not only methodological factors but also biological variability within iNPH populations. iNPH populations are clinically heterogeneous, with differences in comorbidity burden, disease duration and underlying neuropathology potentially contributing to divergent cognitive trajectories. In particular, the presence of coexisting cerebrovascular disease or neurodegenerative pathology can independently impair cognition and may attenuate cognitive gains following shunt surgery [8, 9, 18]. These factors should therefore be considered alongside methodological variability when interpreting differences in reported outcomes.

### Evidence for cognitive improvement with appropriate testing

Despite considerable methodological heterogeneity across studies, the meta-analysis demonstrates that shunt surgery is associated with clinically meaningful cognitive improvement when domain-sensitive tests targeting iNPH-related deficits are used, thereby extending a previous meta-analysis of 23 studies that focused on statistically significant change [42]. Among the ten cognitive outcomes assessed in the present review, TMT-A, GPB-D and TMT-B showed statistically significant improvements that exceeded both expected practice effects and all prespecified thresholds for clinically meaningful change [31–35]. Sensitivity analyses confirmed the robustness of these findings.

TMT-A and GPB-D demonstrated low heterogeneity, indicating consistent effects across studies. Although TMT-B showed moderate to substantial heterogeneity, this may partly reflect inconsistent application of the test’s 5-minute discontinuation rule [33], which patients with severe processing speed deficits are more likely to reach, potentially inflating between-study variability. Together, these findings indicate that when validated measures targeting core iNPH cognitive deficits are used, shunt surgery may yield reliable and clinically meaningful cognitive improvement.

### Limitations

Several limitations arise from the nature of the existing iNPH literature. The predominance of pre–post within-subject designs limits causal inference regarding the effects of shunt surgery on cognitive change. Only one study included a healthy control group assessed over a comparable follow-up interval without shunt intervention [43] and only one double-blind placebo-controlled RCT was identified [15]. Study quality was variable, with common limitations including lack of prospective sample size calculation and blinded outcome assessment. Diagnostic criteria for iNPH were not consistently specified across the included studies and iNPH populations themselves are clinically heterogeneous, with differences in comorbidity burden, disease duration, underlying neuropathology and surgical selection criteria potentially contributing to variability in reported cognitive outcomes [8, 9]. Future prospective studies incorporating appropriate comparison groups and standardised domain-sensitive cognitive tests will therefore be important for more clearly isolating treatment-related effects.

Further limitations relate to heterogeneity in cognitive measurement. Studies varied widely in the cognitive tests employed, timing of post-operative assessments and definitions of improvement, limiting comparability and generalisability across studies. Reliable change indices were not reported in the included literature, which may have provided additional insight into clinically meaningful individual-level change.

Meta-analysis of several commonly used executive function and processing speed tests (Letter Fluency, Stroop, SDMT, Digit Symbol/Coding) was not possible due to insufficient reporting of relevant summary statistics and heterogeneity in test versions or scoring methods. The GPB-D meta-analysis was limited by the small number of extractable datasets, although effect direction and magnitude were consistent across studies. A small number of studies reported electronically administered cognitive assessments, most commonly heterogeneous, non-standardised reaction-time tasks, precluding pooled analysis. Digitally administered cognitive assessments may become increasingly relevant as technological innovations and scalable cognitive testing approaches become more widely integrated into healthcare. In addition, restricting the search to English-language publications may have introduced geographical bias.

Despite these limitations, this review represents the most comprehensive synthesis to date of cognitive assessment methods and post-surgical outcomes in iNPH, identifying key methodological factors that have systematically influenced outcome reporting in this field.

### Clinical practice implications

Although cognitive impairment has been cited as the least responsive symptom of Hakim’s triad following shunt surgery [16], the present review suggests that previously inconsistent findings may partly reflect methodological limitations in cognitive assessment rather than true treatment ineffectiveness. In particular, more than half of studies failed to assess domains most relevant to iNPH or relied on global screening tools with limited sensitivity to change. Together with the characteristic frontal–subcortical cognitive profile and frequent comorbidity observed in iNPH populations [3, 4], these findings support a minimal domain-sensitive battery incorporating two complementary components to assess baseline impairment and treatment response.

First, brief tests sensitive to frontal–subcortical dysfunction should be included when evaluating baseline impairment and post-shunt response. In practice, this may involve selecting at least one measure of processing and/or psychomotor speed and one measure of executive function. In the present meta-analyses, validated measures such as TMT-A (processing and psychomotor speed), GPB-D (psychomotor speed) and TMT-B (executive function) demonstrated statistically significant and clinically meaningful improvement. These measures are brief, require minimal equipment and have well-established normative data, making them practical for use in multidisciplinary clinical settings. Other tests sensitive to frontal–subcortical dysfunction (Letter Fluency, Stroop, SDMT, Coding or Symbol Search) may represent reasonable alternatives, although these could not be meta-analysed in the present review. Importantly, these tests have age-adjusted normative data, supporting reliable interpretation of change relative to expected performance, enabling more accurate detection of clinically meaningful improvement across a range of premorbid ability levels and reducing susceptibility to ceiling effects.

Second, a global screening measure (MoCA, ACE-III or MMSE) may be included to evaluate overall cognitive status, support differential diagnosis and identify cognitive comorbidities that may influence outcomes, rather than as primary measures of treatment-related cognitive change. Common iNPH comorbidities such as Alzheimer’s disease or vascular pathology can independently impair cognition and attenuate post-shunt cognitive improvement, making their detection important for accurately interpreting treatment outcomes [17–19]. The MoCA and ACE-III, which include broader domain coverage than the MMSE, may provide greater sensitivity for this purpose [44, 45]. Interpreting performance on these measures alongside the clinical presentation may help distinguish iNPH from common differentials, such as Alzheimer’s disease. For example, in iNPH, slowed processing speed and executive dysfunction may secondarily affect memory encoding and retrieval, whereas Alzheimer’s disease is more typically characterised by prominent episodic memory impairment and orientation difficulties, although overlap may occur in patients with mixed pathology [4, 14, 46].

However, dementia screening measures appear less sensitive to treatment-related cognitive change in iNPH and therefore should not be relied upon in isolation when evaluating shunt response. This may partly explain why some studies, including a recent RCT using the MoCA as the primary cognitive outcome, have reported limited cognitive improvement following shunt surgery [15].

While the minimal battery described above may be sufficient in many cases, more comprehensive neuropsychological assessment may be informative in selected situations. For example, this may be particularly relevant in patients with high premorbid ability, where cognitive difficulties appear subtle, or where there is concern about comorbid neurodegenerative pathology that may not be adequately captured on brief screening measures.

More broadly, the findings of this review highlight the need for greater standardisation of cognitive assessment in iNPH. Consistent use of domain-relevant tests alongside structured approaches for defining clinically meaningful change has the potential to improve diagnostic accuracy, evaluation of shunt response and long-term management of patients with iNPH, and may also facilitate the development of standardised cognitive endpoints for future clinical trials.

## Conclusion

This systematic review demonstrates that when cognitive assessment tools sensitive to frontal–subcortical dysfunction are used, there is evidence of clinically meaningful post-shunt cognitive improvement in iNPH. Much of the inconsistency in the prior literature reflects methodological heterogeneity in cognitive assessment and definitions of improvement, rather than treatment ineffectiveness. Many commonly used tests fail to capture the core cognitive deficits observed in iNPH, limiting their utility for assessing baseline impairment and post-shunt change.

These findings emphasise the need for standardised, iNPH-appropriate cognitive evaluation tools that are feasible for routine practice and incorporate validated metrics for detecting clinically relevant cognitive improvement. Future research should prioritise the development and validation of such tools, including measures of processing speed, psychomotor speed and executive function, with longitudinal follow-up to capture the trajectory of post-shunt cognitive recovery. Importantly, standardisation of cognitive assessment has implications not only for clinical evaluation of shunt response but also for the selection of robust cognitive endpoints in future interventional trials.

## Supplementary Information

Below is the link to the electronic supplementary material.

Supplementary Material 1

## Data Availability

All data analysed in this study were derived from previously published studies and are cited in the manuscript and/or supplementary materials.
