# Supplementary material for: Cognitive assessment methods and outcomes following shunt surgery in idiopathic normal pressure hydrocephalus (iNPH): a systematic review and meta-analysis
Source: Fluids Barriers CNS. 2026 May 11;23:70. doi: 10.1186/s12987-026-00808-3 (PMC13169649; doi:10.1186/s12987-026-00808-3)
Supplement: Supplementary file 1 — Supplementary Material 1 [file 12987_2026_808_MOESM1_ESM.docx]

**Supplementary Materials**

Cognitive assessment methods and outcomes following shunt surgery in idiopathic normal pressure hydrocephalus (iNPH): a systematic review and meta-analysis

| **Supplementary Table 1.** Domain-level MINORS quality assessment ratings for the 195 included studies. | | | | | | | | | |
| --- | --- | --- | --- | --- | --- | --- | --- | --- | --- |
| **Study**  **first author** | **Study year** | **Study**  **aim** | **Selection bias (Consecutive patients)** | **Prospective study design** | **Appropriate data collection methods** | **Unbiased assessment** | **Adequate follow up period** | **Attrition (Loss to follow-up)** | **Prospective sample size calculation** |
| Abu Hamdeh | 2018 | 1 | 1 | 2 | 2 | 1 | 2 | 2 | 0 |
| Acosta | 2021 | 1 | 0 | 1 | 0 | 0 | 1 | 2 | 0 |
| Agerskov | 2018 | 2 | 2 | 1 | 1 | 1 | 2 | 1 | 0 |
| Akiba | 2018 | 2 | 2 | 1 | 2 | 0 | 2 | 0 | 0 |
| Akiguchi | 2008 | 1 | 0 | 1 | 2 | 1 | 1 | 2 | 0 |
| Andrén | 2014 | 2 | 2 | 1 | 2 | 0 | 2 | 1 | 0 |
| Andrén | 2024 | 1 | 2 | 2 | 2 | 1 | 1 | 1 | 0 |
| Asahara | 2023 | 2 | 1 | 1 | 2 | 0 | 1 | 0 | 0 |
| Aygok | 2005 | 2 | 2 | 2 | 1 | 0 | 2 | 1 | 0 |
| Behrens | 2020 | 2 | 2 | 2 | 2 | 1 | 2 | 1 | 0 |
| Belotti | 2022 | 1 | 1 | 1 | 0 | 0 | 2 | 1 | 0 |
| Bloch | 2012 | 1 | 2 | 1 | 0 | 0 | 1 | 2 | 0 |
| Broggi | 2016 | 1 | 1 | 2 | 2 | 0 | 2 | 1 | 0 |
| Bubeníková | 2025 | 1 | 2 | 2 | 2 | 1 | 2 | 2 | 0 |
| Bugalho | 2013 | 1 | 1 | 1 | 1 | 0 | 1 | 1 | 0 |
| Büyükgök | 2021 | 2 | 1 | 2 | 2 | 2 | 2 | 0 | 0 |
| Cage | 2011 | 1 | 0 | 1 | 0 | 0 | 1 | 0 | 0 |
| Calcagni | 2012 | 1 | 0 | 2 | 1 | 0 | 1 | 1 | 0 |
| Caruso | 2024 | 1 | 1 | 2 | 0 | 0 | 2 | 0 | 0 |
| Chang | 2006 | 2 | 2 | 2 | 2 | 1 | 2 | 2 | 0 |
| Chaudhry | 2007 | 2 | 1 | 2 | 2 | 1 | 2 | 2 | 0 |
| Chen | 1994 | 1 | 2 | 2 | 1 | 0 | 2 | 2 | 0 |
| **Note.** MINORS items were scored 0–2 (higher scores indicate stronger methodological features). Item definitions: Study aim = cognitive outcomes explicitly stated as an objective; Selection bias = consecutive patients; Prospective design = prospective data collection; Data collection = appropriate cognitive measures; Unbiased assessment = independent/blinded assessment of outcome; Adequate follow-up = duration appropriate for outcomes; Attrition = low loss to follow-up; Sample size = a priori calculation. | | | | | | | | | |
| **Supplementary Table 1.** (Continued) | | | |  |  |  |  |  |  |
| **Study**  **first author** | **Study year** | **Study**  **aim** | **Selection bias (Consecutive patients)** | **Prospective study design** | **Appropriate data collection methods** | **Unbiased assessment** | **Adequate follow up period** | **Attrition (Loss to follow-up)** | **Prospective sample size calculation** |
| Chen | 2022 | 1 | 2 | 1 | 0 | 0 | 2 | 1 | 0 |
| Chen | 2025 | 1 | 2 | 1 | 1 | 0 | 2 | 0 | 0 |
| Chiaravalloti | 2020 | 1 | 1 | 1 | 2 | 0 | 2 | 2 | 0 |
| Chidiac | 2022 | 1 | 2 | 2 | 1 | 0 | 2 | 0 | 0 |
| Craven | 2016 | 1 | 1 | 1 | 1 | 0 | 2 | 1 | 0 |
| Damasceno | 1997 | 1 | 1 | 1 | 2 | 1 | 2 | 2 | 0 |
| De Oliveira | 2013 | 1 | 1 | 2 | 1 | 0 | 2 | 2 | 0 |
| De Oliveira | 2021 | 1 | 2 | 2 | 1 | 0 | 2 | 0 | 0 |
| del Mar Matarín | 2007 | 1 | 1 | 2 | 2 | 1 | 2 | 2 | 0 |
| Di Renzo | 2022 | 1 | 0 | 1 | 2 | 0 | 2 | 2 | 0 |
| Dixon | 2002 | 1 | 0 | 1 | 0 | 0 | 1 | 2 | 0 |
| Duinkerke | 2004 | 2 | 1 | 2 | 2 | 1 | 2 | 2 | 0 |
| Eleftheriou | 2018 | 1 | 2 | 2 | 1 | 0 | 2 | 0 | 0 |
| Eleftheriou | 2020 | 1 | 2 | 2 | 2 | 1 | 2 | 2 | 0 |
| Fang | 2022 | 1 | 2 | 1 | 1 | 0 | 2 | 2 | 0 |
| Farahmand | 2016 | 1 | 2 | 2 | 2 | 2 | 2 | 2 | 0 |
| Foss | 2007 | 2 | 2 | 1 | 2 | 0 | 2 | 2 | 0 |
| Gago | 2022 | 1 | 2 | 2 | 1 | 0 | 2 | 1 | 0 |
| Giannini | 2019 | 1 | 2 | 2 | 2 | 1 | 2 | 0 | 0 |
| Gleichgerrcht | 2009 | 2 | 1 | 2 | 2 | 1 | 2 | 2 | 0 |
| Goertz | 2024 | 1 | 2 | 2 | 2 | 0 | 2 | 0 | 0 |
| Gold | 2023 | 2 | 2 | 2 | 2 | 1 | 2 | 1 | 1 |
| **Note.** MINORS items were scored 0–2 (higher scores indicate stronger methodological features). Item definitions: Study aim = cognitive outcomes explicitly stated as an objective; Selection bias = consecutive patients; Prospective design = prospective data collection; Data collection = appropriate cognitive measures; Unbiased assessment = independent/blinded assessment of outcome; Adequate follow-up = duration appropriate for outcomes; Attrition = low loss to follow-up; Sample size = a priori calculation. | | | | | | | | | |
| **Supplementary Table 1.** (Continued) | | | |  |  |  |  |  |  |
| **Study**  **first author** | **Study year** | **Study**  **aim** | **Selection bias (Consecutive patients)** | **Prospective study design** | **Appropriate data collection methods** | **Unbiased assessment** | **Adequate follow up period** | **Attrition (Loss to follow-up)** | **Prospective sample size calculation** |
| Golomb | 2000 | 2 | 0 | 2 | 2 | 1 | 1 | 0 | 0 |
| Graff-Radford | 1986 | 1 | 1 | 2 | 1 | 0 | 2 | 2 | 0 |
| Grasso | 2019 | 1 | 2 | 1 | 1 | 0 | 2 | 1 | 0 |
| Grasso | 2023 | 1 | 2 | 1 | 1 | 0 | 2 | 2 | 0 |
| Hallqvist | 2022 | 2 | 2 | 1 | 1 | 0 | 2 | 2 | 0 |
| Hamilton | 2010 | 2 | 0 | 2 | 2 | 1 | 2 | 1 | 0 |
| Hashimoto | 2010 | 1 | 2 | 2 | 1 | 0 | 2 | 2 | 1 |
| Hasselbalch | 2023 | 1 | 1 | 1 | 1 | 0 | 1 | 2 | 0 |
| He | 2022 | 1 | 2 | 2 | 1 | 0 | 2 | 0 | 0 |
| Hellström | 2008 | 2 | 2 | 2 | 2 | 1 | 2 | 1 | 0 |
| Hellström | 2012 | 2 | 2 | 2 | 2 | 1 | 2 | 1 | 0 |
| Hiraoka | 2010 | 1 | 0 | 2 | 2 | 0 | 2 | 0 | 0 |
| Hiraoka | 2015 | 1 | 1 | 2 | 2 | 1 | 2 | 2 | 0 |
| Hong | 2018 | 1 | 1 | 2 | 1 | 0 | 2 | 1 | 0 |
| Huang | 2022 | 1 | 1 | 1 | 1 | 0 | 1 | 2 | 0 |
| Huang | 2024 | 1 | 1 | 1 | 1 | 0 | 2 | 2 | 0 |
| Hülser | 2022 | 2 | 2 | 2 | 2 | 1 | 2 | 2 | 0 |
| Iddon | 1999 | 2 | 1 | 2 | 2 | 1 | 2 | 0 | 0 |
| Illán-Gala | 2017 | 1 | 1 | 2 | 1 | 1 | 2 | 2 | 0 |
| Ishikawa | 2021 | 0 | 2 | 2 | 1 | 0 | 2 | 2 | 0 |
| Ishikawa | 2023 | 2 | 1 | 2 | 2 | 0 | 2 | 2 | 0 |
| Jingami | 2019 | 1 | 2 | 2 | 1 | 0 | 1 | 2 | 0 |
| **Note.** MINORS items were scored 0–2 (higher scores indicate stronger methodological features). Item definitions: Study aim = cognitive outcomes explicitly stated as an objective; Selection bias = consecutive patients; Prospective design = prospective data collection; Data collection = appropriate cognitive measures; Unbiased assessment = independent/blinded assessment of outcome; Adequate follow-up = duration appropriate for outcomes; Attrition = low loss to follow-up; Sample size = a priori calculation. | | | | | | | | | |
| **Supplementary Table 1.** (Continued) | | | |  |  |  |  |  |  |
| **Study**  **first author** | **Study year** | **Study**  **aim** | **Selection bias (Consecutive patients)** | **Prospective study design** | **Appropriate data collection methods** | **Unbiased assessment** | **Adequate follow up period** | **Attrition (Loss to follow-up)** | **Prospective sample size calculation** |
| Junkkari | 2017 | 0 | 2 | 2 | 1 | 0 | 2 | 0 | 0 |
| Kajimoto | 2022 | 2 | 1 | 1 | 2 | 0 | 2 | 2 | 0 |
| Kambara | 2021 | 2 | 2 | 1 | 1 | 0 | 2 | 0 | 0 |
| Kamohara | 2020 | 2 | 2 | 2 | 2 | 1 | 2 | 2 | 0 |
| Kanemoto | 2016 | 1 | 1 | 2 | 2 | 0 | 2 | 2 | 0 |
| Kanemoto | 2019 | 1 | 1 | 1 | 2 | 1 | 2 | 0 | 0 |
| Kanno | 2017 | 1 | 2 | 2 | 2 | 0 | 2 | 0 | 0 |
| Kanno | 2021 | 2 | 2 | 2 | 2 | 0 | 2 | 2 | 0 |
| Katzen | 2011 | 2 | 1 | 2 | 2 | 1 | 2 | 2 | 0 |
| Kazui | 2011 | 1 | 2 | 2 | 1 | 0 | 2 | 1 | 0 |
| Kazui | 2013 | 1 | 2 | 2 | 1 | 0 | 2 | 2 | 0 |
| Kazui | 2015 | 1 | 2 | 2 | 2 | 1 | 2 | 1 | 1 |
| Kazui | 2016 | 1 | 1 | 2 | 2 | 1 | 2 | 1 | 0 |
| Kilinç | 2022 | 0 | 2 | 1 | 1 | 0 | 2 | 2 | 0 |
| Kito | 2009 | 0 | 2 | 2 | 1 | 0 | 1 | 1 | 0 |
| Klassen | 2011 | 1 | 1 | 1 | 0 | 0 | 2 | 1 | 0 |
| Klinge | 2012 | 2 | 2 | 2 | 2 | 1 | 2 | 1 | 0 |
| Korhonen | 2019 | 0 | 1 | 1 | 1 | 1 | 2 | 2 | 0 |
| Krahulik | 2020 | 2 | 2 | 2 | 1 | 0 | 2 | 2 | 0 |
| Krauss | 1996 | 1 | 1 | 2 | 0 | 0 | 1 | 2 | 0 |
| Krauss | 1997 | 1 | 1 | 2 | 0 | 0 | 1 | 2 | 0 |
| Lee | 2021 | 1 | 2 | 2 | 1 | 0 | 2 | 2 | 0 |
| **Note.** MINORS items were scored 0–2 (higher scores indicate stronger methodological features). Item definitions: Study aim = cognitive outcomes explicitly stated as an objective; Selection bias = consecutive patients; Prospective design = prospective data collection; Data collection = appropriate cognitive measures; Unbiased assessment = independent/blinded assessment of outcome; Adequate follow-up = duration appropriate for outcomes; Attrition = low loss to follow-up; Sample size = a priori calculation. | | | | | | | | | |
| **Supplementary Table 1.** (Continued) | | | |  |  |  |  |  |  |
| **Study**  **first author** | **Study year** | **Study**  **aim** | **Selection bias (Consecutive patients)** | **Prospective study design** | **Appropriate data collection methods** | **Unbiased assessment** | **Adequate follow up period** | **Attrition (Loss to follow-up)** | **Prospective sample size calculation** |
| Liouta | 2017 | 2 | 2 | 2 | 2 | 1 | 1 | 1 | 0 |
| Liu | 2016 | 1 | 2 | 1 | 1 | 0 | 1 | 2 | 0 |
| Liu | 2020 | 1 | 2 | 1 | 1 | 0 | 2 | 0 | 0 |
| Luciano | 2023 | 1 | 0 | 2 | 2 | 1 | 2 | 2 | 0 |
| Luciano | 2025 | 1 | 0 | 2 | 2 | 1 | 2 | 2 | 1 |
| Lundin | 2013a | 0 | 2 | 2 | 1 | 0 | 2 | 0 | 1 |
| Lundin | 2013b | 0 | 2 | 2 | 1 | 0 | 1 | 0 | 0 |
| Ma | 2017 | 1 | 2 | 1 | 0 | 0 | 2 | 2 | 0 |
| Macki | 2020 | 1 | 1 | 2 | 1 | 0 | 1 | 2 | 0 |
| Malem | 2014 | 0 | 1 | 1 | 1 | 0 | 1 | 2 | 0 |
| Malm | 1995a | 2 | 2 | 2 | 1 | 1 | 2 | 2 | 0 |
| Malm | 1995b | 1 | 2 | 2 | 1 | 0 | 2 | 2 | 0 |
| Malm | 2000 | 1 | 1 | 2 | 1 | 0 | 2 | 0 | 0 |
| Mataro | 2003 | 2 | 1 | 2 | 2 | 1 | 2 | 2 | 0 |
| Mataro | 2007 | 2 | 2 | 2 | 2 | 1 | 2 | 1 | 0 |
| Matsuoka | 2022 | 0 | 2 | 1 | 1 | 0 | 1 | 2 | 0 |
| McGovern | 2019 | 2 | 2 | 2 | 2 | 1 | 2 | 2 | 0 |
| McGrath | 2024 | 1 | 1 | 1 | 0 | 1 | 2 | 1 | 0 |
| Messerer | 2022 | 0 | 1 | 1 | 0 | 0 | 2 | 1 | 0 |
| Miyajima | 2013 | 1 | 2 | 2 | 2 | 0 | 2 | 2 | 0 |
| Miyajima | 2016 | 1 | 2 | 2 | 1 | 0 | 2 | 2 | 0 |
| Mori | 2001 | 0 | 2 | 1 | 0 | 0 | 2 | 1 | 0 |
| **Note.** MINORS items were scored 0–2 (higher scores indicate stronger methodological features). Item definitions: Study aim = cognitive outcomes explicitly stated as an objective; Selection bias = consecutive patients; Prospective design = prospective data collection; Data collection = appropriate cognitive measures; Unbiased assessment = independent/blinded assessment of outcome; Adequate follow-up = duration appropriate for outcomes; Attrition = low loss to follow-up; Sample size = a priori calculation. | | | | | | | | | |
| **Supplementary Table 1.** (Continued) | | | |  |  |  |  |  |  |
| **Study**  **first author** | **Study year** | **Study**  **aim** | **Selection bias (Consecutive patients)** | **Prospective study design** | **Appropriate data collection methods** | **Unbiased assessment** | **Adequate follow up period** | **Attrition (Loss to follow-up)** | **Prospective sample size calculation** |
| Moriya | 2015 | 1 | 1 | 2 | 2 | 1 | 2 | 2 | 0 |
| Mostile | 2021 | 0 | 1 | 1 | 2 | 1 | 1 | 0 | 0 |
| Murakami | 2018 | 1 | 1 | 2 | 2 | 0 | 1 | 2 | 0 |
| Nakajima | 2011 | 1 | 2 | 2 | 2 | 0 | 2 | 2 | 0 |
| Nakajima | 2015a | 2 | 2 | 1 | 2 | 1 | 2 | 2 | 0 |
| Nakajima | 2015b | 2 | 1 | 2 | 2 | 1 | 2 | 0 | 0 |
| Nakajima | 2018a | 1 | 2 | 1 | 2 | 0 | 2 | 1 | 0 |
| Nakajima | 2018b | 2 | 1 | 1 | 2 | 0 | 2 | 2 | 0 |
| Nakajima | 2021 | 2 | 2 | 1 | 2 | 1 | 2 | 1 | 0 |
| Nakatsu | 2016 | 2 | 1 | 1 | 0 | 0 | 2 | 1 | 0 |
| Nakayama | 2007 | 1 | 0 | 2 | 1 | 0 | 1 | 2 | 0 |
| Narita | 2016 | 1 | 2 | 1 | 1 | 0 | 2 | 2 | 0 |
| Oike | 2021 | 0 | 2 | 1 | 1 | 0 | 1 | 1 | 0 |
| Patel | 2012 | 2 | 1 | 2 | 2 | 1 | 2 | 0 | 0 |
| Pesce | 2022 | 2 | 1 | 1 | 2 | 0 | 2 | 2 | 0 |
| Petersen | 2014 | 1 | 2 | 2 | 2 | 0 | 2 | 2 | 0 |
| Peterson | 2016 | 2 | 2 | 1 | 1 | 1 | 2 | 2 | 0 |
| Peterson | 2019 | 2 | 1 | 2 | 1 | 1 | 2 | 1 | 0 |
| Pfisterer | 2007 | 1 | 2 | 2 | 0 | 0 | 1 | 2 | 0 |
| Pinto | 2013 | 1 | 0 | 2 | 1 | 0 | 2 | 0 | 1 |
| Poca | 2004 | 2 | 2 | 2 | 2 | 1 | 2 | 2 | 0 |
| Poca | 2005 | 2 | 2 | 1 | 2 | 1 | 2 | 2 | 0 |
| **Note.** MINORS items were scored 0–2 (higher scores indicate stronger methodological features). Item definitions: Study aim = cognitive outcomes explicitly stated as an objective; Selection bias = consecutive patients; Prospective design = prospective data collection; Data collection = appropriate cognitive measures; Unbiased assessment = independent/blinded assessment of outcome; Adequate follow-up = duration appropriate for outcomes; Attrition = low loss to follow-up; Sample size = a priori calculation. | | | | | | | | | |
| **Supplementary Table 1.** (Continued) | | | |  |  |  |  |  |  |
| **Study**  **first author** | **Study year** | **Study**  **aim** | **Selection bias (Consecutive patients)** | **Prospective study design** | **Appropriate data collection methods** | **Unbiased assessment** | **Adequate follow up period** | **Attrition (Loss to follow-up)** | **Prospective sample size calculation** |
| Poca | 2012 | 1 | 2 | 2 | 2 | 0 | 2 | 2 | 0 |
| Pujari | 2008 | 1 | 2 | 1 | 1 | 0 | 2 | 0 | 0 |
| Raftopoulos | 1994 | 2 | 2 | 2 | 1 | 1 | 2 | 2 | 0 |
| Raneri | 2017 | 0 | 2 | 1 | 0 | 0 | 2 | 2 | 0 |
| Razay | 2009 | 1 | 2 | 2 | 1 | 0 | 2 | 1 | 0 |
| Razay | 2019 | 1 | 2 | 2 | 1 | 0 | 2 | 1 | 2 |
| Rydja | 2021a | 0 | 2 | 2 | 2 | 1 | 2 | 0 | 0 |
| Rydja | 2021b | 0 | 2 | 1 | 2 | 2 | 1 | 1 | 0 |
| Saadaldeen | 2025 | 2 | 2 | 1 | 2 | 1 | 1 | 1 | 0 |
| Saito | 2011 | 2 | 1 | 2 | 2 | 1 | 2 | 1 | 0 |
| Saito | 2020 | 2 | 1 | 1 | 2 | 1 | 1 | 2 | 0 |
| Sakurai | 2022 | 1 | 1 | 1 | 1 | 0 | 2 | 2 | 0 |
| Sand | 1994 | 0 | 2 | 2 | 1 | 1 | 1 | 1 | 0 |
| Savolainen | 2002 | 1 | 2 | 2 | 2 | 0 | 2 | 0 | 0 |
| Shanks | 2019 | 1 | 2 | 2 | 1 | 0 | 2 | 1 | 0 |
| Shaw | 2016 | 2 | 2 | 1 | 1 | 0 | 2 | 1 | 0 |
| Shinoda | 2017 | 1 | 2 | 1 | 2 | 1 | 2 | 1 | 0 |
| Sindorio | 2017 | 2 | 0 | 2 | 2 | 1 | 2 | 0 | 0 |
| Sirkka | 2021 | 1 | 2 | 2 | 2 | 1 | 2 | 1 | 0 |
| Skalický | 2022 | 1 | 1 | 2 | 2 | 1 | 2 | 0 | 0 |
| Snöbohm | 2022 | 0 | 2 | 1 | 1 | 0 | 2 | 2 | 0 |
| Solana | 2012 | 2 | 2 | 2 | 2 | 0 | 2 | 1 | 0 |
| **Note.** MINORS items were scored 0–2 (higher scores indicate stronger methodological features). Item definitions: Study aim = cognitive outcomes explicitly stated as an objective; Selection bias = consecutive patients; Prospective design = prospective data collection; Data collection = appropriate cognitive measures; Unbiased assessment = independent/blinded assessment of outcome; Adequate follow-up = duration appropriate for outcomes; Attrition = low loss to follow-up; Sample size = a priori calculation. | | | | | | | | | |
| **Supplementary Table 1.** (Continued) | | | |  |  |  |  |  |  |
| **Study**  **first author** | **Study year** | **Study**  **aim** | **Selection bias (Consecutive patients)** | **Prospective study design** | **Appropriate data collection methods** | **Unbiased assessment** | **Adequate follow up period** | **Attrition (Loss to follow-up)** | **Prospective sample size calculation** |
| Sorteberg | 2004 | 1 | 2 | 2 | 0 | 1 | 2 | 1 | 0 |
| Spagnoli | 2006 | 1 | 2 | 2 | 0 | 0 | 2 | 2 | 0 |
| Spanu | 1989 | 1 | 2 | 1 | 1 | 0 | 2 | 2 | 0 |
| Spielmann | 2024 | 2 | 2 | 2 | 2 | 1 | 2 | 1 | 0 |
| St. Louis | 2014 | 1 | 0 | 1 | 2 | 1 | 2 | 0 | 0 |
| Subramanian | 2018 | 0 | 2 | 1 | 0 | 1 | 1 | 2 | 0 |
| Subramanian | 2021 | 1 | 2 | 1 | 0 | 0 | 1 | 0 | 0 |
| Sundström | 2016 | 1 | 2 | 1 | 1 | 0 | 2 | 0 | 0 |
| Takeuchi | 2019 | 1 | 2 | 1 | 1 | 0 | 2 | 2 | 0 |
| Thomas | 2005 | 2 | 1 | 1 | 2 | 1 | 2 | 0 | 0 |
| Thompson | 2017 | 1 | 1 | 1 | 1 | 1 | 1 | 1 | 0 |
| Thomsen | 1986 | 2 | 2 | 2 | 2 | 0 | 2 | 0 | 0 |
| Tisell | 2003 | 1 | 2 | 2 | 1 | 1 | 2 | 2 | 0 |
| Tisell | 2011 | 2 | 2 | 2 | 2 | 2 | 2 | 2 | 0 |
| Todisco | 2020 | 1 | 2 | 2 | 2 | 2 | 2 | 2 | 0 |
| Tominaga | 2023 | 1 | 2 | 1 | 1 | 0 | 1 | 1 | 0 |
| Trevisi | 2021 | 0 | 2 | 1 | 0 | 0 | 2 | 1 | 0 |
| Tsakanikas | 2009 | 0 | 1 | 2 | 2 | 0 | 2 | 0 | 0 |
| Tseng | 2023 | 2 | 2 | 2 | 0 | 0 | 2 | 0 | 0 |
| Valsecchi | 2022 | 0 | 1 | 2 | 0 | 0 | 2 | 1 | 0 |
| Virhammar | 2014 | 0 | 2 | 1 | 1 | 0 | 2 | 0 | 0 |
| Virhammar | 2020 | 0 | 1 | 2 | 1 | 1 | 2 | 1 | 0 |
| **Note.** MINORS items were scored 0–2 (higher scores indicate stronger methodological features). Item definitions: Study aim = cognitive outcomes explicitly stated as an objective; Selection bias = consecutive patients; Prospective design = prospective data collection; Data collection = appropriate cognitive measures; Unbiased assessment = independent/blinded assessment of outcome; Adequate follow-up = duration appropriate for outcomes; Attrition = low loss to follow-up; Sample size = a priori calculation. | | | | | | | | | |
| **Supplementary Table 1.** (Continued) | | | |  |  |  |  |  |  |
| **Study**  **first author** | **Study year** | **Study**  **aim** | **Selection bias (Consecutive patients)** | **Prospective study design** | **Appropriate data collection methods** | **Unbiased assessment** | **Adequate follow up period** | **Attrition (Loss to follow-up)** | **Prospective sample size calculation** |
| Vivas-Buitrago | 2020 | 0 | 2 | 1 | 0 | 0 | 1 | 2 | 0 |
| Vorstrup | 1987 | 1 | 0 | 2 | 1 | 0 | 2 | 1 | 0 |
| Wada | 2013 | 2 | 0 | 1 | 2 | 0 | 2 | 2 | 0 |
| Wesner | 2022 | 2 | 2 | 1 | 1 | 0 | 1 | 0 | 0 |
| Wetzel | 2018 | 1 | 0 | 2 | 2 | 1 | 2 | 2 | 0 |
| Wikkelsø | 1986 | 2 | 2 | 2 | 1 | 1 | 2 | 1 | 0 |
| Wikkelsø | 2013 | 1 | 2 | 2 | 2 | 0 | 2 | 2 | 0 |
| Williams | 2022 | 2 | 1 | 2 | 2 | 1 | 2 | 0 | 0 |
| Wolfsegger | 2021 | 1 | 1 | 1 | 2 | 1 | 2 | 2 | 0 |
| Wu | 2021 | 1 | 1 | 1 | 1 | 0 | 1 | 1 | 0 |
| Yamada | 2013 | 1 | 1 | 2 | 1 | 0 | 2 | 2 | 0 |
| Yamada | 2017 | 0 | 2 | 2 | 0 | 0 | 2 | 2 | 0 |
| Yamamoto | 2013 | 2 | 0 | 2 | 2 | 0 | 2 | 2 | 0 |
| Yang | 2016 | 1 | 1 | 2 | 0 | 0 | 1 | 2 | 0 |
| Yang | 2017 | 1 | 2 | 1 | 0 | 1 | 2 | 2 | 0 |
| Yang | 2023 | 0 | 1 | 2 | 0 | 0 | 2 | 2 | 0 |
| Yang | 2025 | 2 | 1 | 2 | 1 | 0 | 2 | 0 | 0 |
| Yasar | 2017 | 1 | 2 | 2 | 1 | 1 | 1 | 1 | 0 |
| Yerneni | 2021 | 1 | 1 | 1 | 0 | 0 | 2 | 1 | 0 |
| **Note.** MINORS items were scored 0–2 (higher scores indicate stronger methodological features). Item definitions: Study aim = cognitive outcomes explicitly stated as an objective; Selection bias = consecutive patients; Prospective design = prospective data collection; Data collection = appropriate cognitive measures; Unbiased assessment = independent/blinded assessment of outcome; Adequate follow-up = duration appropriate for outcomes; Attrition = low loss to follow-up; Sample size = a priori calculation. | | | | | | | | | |

| **Supplementary Table 2.** Full list of cognitive tests reported in the 195 included studies, with reported cognitive outcome data. | | | |
| --- | --- | --- | --- |
| **Cognitive Test** | **Cognitive Domain /**  **Test Type** | **Battery** | **Studies (n)** |
| Alzheimer's Disease Assessment Scale (ADAS) Comprehension of Spoken Language | Language | ADAS | 1 |
| ADAS Constructional Praxis | Visuospatial function | ADAS | 2 |
| ADAS Following Commands | Language | ADAS | 1 |
| ADAS Ideational Praxis | Executive function / Praxis | ADAS | 1 |
| ADAS Naming Objects and Fingers | Language | ADAS | 1 |
| ADAS Orientation | Orientation | ADAS | 1 |
| ADAS Remembering Test Instructions | Memory | ADAS | 1 |
| ADAS Spoken Language Ability | Language | ADAS | 1 |
| ADAS Word Recall | Memory | ADAS | 2 |
| ADAS Word Recognition | Memory | ADAS | 2 |
| ADAS Word-Finding Difficulty | Language | ADAS | 1 |
| Addenbrooke's Cognitive Exam (ACE) | Dementia screen |  | 2 |
| Alphabet Writing (Timed, Non-standardised Test) | Psychomotor speed |  | 3 |
| Attention (Unspecified Test) | Attention |  | 1 |
| Barrage Test | Attention |  | 1 |
| Benton Face Recognition | Face recognition / Visual perception |  | 1 |
| Benton Judgement of Line Orientation | Visuospatial function |  | 1 |
| Benton Visual Retention Test | Memory |  | 1 |
| Bingley's Memory Test | Memory |  | 5 |
| Brief Mental Deterioration Battery (BMDB) Word List | Memory | BMDB | 1 |
| **Supplementary Table 2.** (Continued) | | | |
| **Cognitive Test** | **Cognitive Domain /**  **Test Type** | **Battery** | **Studies (n)** |
| BMDB Analogies | Executive function | BMDB | 1 |
| BMDB Barrage Test | Attention | BMDB | 1 |
| BMDB Visual Memory - Immediate Recall | Memory | BMDB | 1 |
| Boston Diagnostic Aphasia Examination (BDAS) Fluency | Language |  | 1 |
| Boston Naming Test | Language |  | 7 |
| Buschke-Fuld 10-Word List - Learning and Recall | Memory |  | 1 |
| Cambridge Neuropsychological Test Automated Battery (CANTAB) Intra-Extra Dimensional Set Shift | Executive function | CANTAB | 1 |
| CANTAB Pattern Recognition | Memory | CANTAB | 1 |
| CANTAB Spatial Recognition | Memory | CANTAB | 1 |
| CANTAB Spatial Span | Working memory | CANTAB | 1 |
| Consortium to Establish a Registry for Alzheimer’s Disease (CERAD) Boston Naming Test | Language | CERAD | 2 |
| CERAD Constructional Praxis | Visuospatial function | CERAD | 2 |
| CERAD Constructional Praxis - Delayed Recall | Memory | CERAD | 2 |
| CERAD Semantic Fluency | Language / Verbal fluency | CERAD | 2 |
| CERAD Word List (10-item) Learning, Recall and Recognition | Memory | CERAD | 2 |
| Clock Copy Test | Visuospatial function |  | 1 |
| Clock Drawing Test | Visuospatial function |  | 4 |
| Computerized General Neuropsychological iNPH Test (CoGNIT) Choice Reaction Time | Reaction time | CoGNIT | 1 |
| CoGNIT Finger Tapping | Motor skill | CoGNIT | 1 |
| **Supplementary Table 2.** (Continued) | | | |
| **Cognitive Test** | **Cognitive Domain /**  **Test Type** | **Battery** | **Studies (n)** |
| CoGNIT Stroop Congruent | Processing speed | CoGNIT | 1 |
| CoGNIT Stroop Incongruent | Executive function | CoGNIT | 1 |
| CoGNIT Trails A | Processing speed / Psychomotor speed | CoGNIT | 1 |
| CoGNIT Trails B | Executive function | CoGNIT | 1 |
| CoGNIT Word List Learning, Delayed Recall and Recognition | Memory | CoGNIT | 1 |
| Copy Drawing with Landmarks Test | Visuospatial function |  | 1 |
| Corsi Block Tapping Test | Working memory |  | 1 |
| Cube Drawing | Visuospatial function |  | 1 |
| Cylinders Test (timed) | Executive function / Psychomotor speed |  | 1 |
| Delis-Kaplan Executive Function System (DKEFS) Colour Word Interference Test | Executive function | DKEFS | 1 |
| Dementia Rating Scale (DRS) Attention | Attention | DRS | 2 |
| DRS Conceptualisation | Executive function | DRS | 2 |
| DRS Construction | Visuospatial function | DRS | 2 |
| DRS Initiation / Perseveration | Executive function | DRS | 2 |
| DRS Memory | Memory | DRS | 2 |
| DemTect | Dementia screen |  | 2 |
| Design Copy (Unspecified Test) | Visuospatial function |  | 1 |
| Episodic Memory (Unspecified Test) | Memory |  | 1 |
| Facial Affect Test | Social cognition |  | 1 |
| Figure Copy (Unspecified Test) | Visuospatial function |  | 1 |
| **Supplementary Table 2.** (Continued) | | | |
| **Cognitive Test** | **Cognitive Domain /**  **Test Type** | **Battery** | **Studies (n)** |
| Finger Tapping Test | Motor speed |  | 6 |
| Frontal Assessment Battery (FAB) | Executive function screen |  | 35 |
| Fuld Object Memory Test | Memory |  | 1 |
| Goldstein and Scheerer Block Design | Executive function / Visuospatial function |  | 1 |
| Grooved Pegboard Test | Psychomotor speed |  | 27 |
| Guild Memory Test - Paragraph Recall | Memory |  | 1 |
| Hasegawa Dementia Rating Scale - Revised | Dementia screen |  | 1 |
| Hopkins Verbal Learning Test - Learning and Delayed Recall | Memory |  | 2 |
| Identical Forms Test | Visuospatial function |  | 5 |
| Intersecting Pentagons Copy | Visuospatial function |  | 1 |
| Judgement of Line Orientation | Visuospatial function |  | 2 |
| Kendrick Object Learning Test (KOLT) | Memory |  | 1 |
| Kohs Test | Visuospatial function |  | 1 |
| Line Tracing (Timed, Non-standardised Test) | Psychomotor speed |  | 7 |
| Luria Hand Sequencing | Executive function / Motor sequencing |  | 1 |
| Mefferd & Moran Perceptual Speed Test | Perceptual speed |  | 1 |
| Memory Recall (Unspecified Test) | Memory |  | 1 |
| Mental Deterioration Battery - Figure Copy | Visuospatial function |  | 1 |
| Mini-Mental State Exam (MMSE) | Dementia screen |  | 128 |
| Montreal Cognitive Assessment (MoCA) | Dementia screen |  | 9 |
| **Supplementary Table 2.** (Continued) | | | |
| **Cognitive Test** | **Cognitive Domain /**  **Test Type** | **Battery** | **Studies (n)** |
| Multiple Features Targets Cancellation | Attention |  | 1 |
| Neuropsychological Assessment Battery (NAB) Auditory Comprehension | Language | NAB | 1 |
| NAB Bill Payment | Language | NAB | 1 |
| NAB Categories | Executive function | NAB | 1 |
| NAB Daily Living Memory - Immediate Recall, Delayed Recall and Recognition | Memory | NAB | 1 |
| NAB Design Construction | Visuospatial function | NAB | 1 |
| NAB Digit Span Backward | Attention | NAB | 1 |
| NAB Digit Span Forward | Attention | NAB | 1 |
| NAB Dots | Attention | NAB | 1 |
| NAB Driving Scenes | Attention | NAB | 1 |
| NAB Figure Drawing Copy | Visuospatial function | NAB | 1 |
| NAB Figure Recall | Memory | NAB | 1 |
| NAB Judgement | Executive function | NAB | 1 |
| NAB List Learning - Immediate Recall, Delayed Recall, Recognition | Memory | NAB | 1 |
| NAB Map Reading | Visuospatial function | NAB | 1 |
| NAB Mazes | Executive function | NAB | 1 |
| NAB Naming | Language | NAB | 1 |
| NAB Number and Letters | Attention | NAB | 1 |
| NAB Oral Production | Language | NAB | 1 |
| NAB Orientation | Orientation | NAB | 1 |
| **Supplementary Table 2.** (Continued) | | | |
| **Cognitive Test** | **Cognitive Domain /**  **Test Type** | **Battery** | **Studies (n)** |
| NAB Reading Comprehension | Language | NAB | 1 |
| NAB Shape Learning - Immediate and Delayed Recognition | Memory | NAB | 1 |
| NAB Story Learning - Immediate and Delayed Recall | Memory | NAB | 1 |
| NAB Visual Discrimination | Visuospatial function | NAB | 1 |
| NAB Word Generation | Executive function | NAB | 1 |
| NAB Writing | Language | NAB | 1 |
| Neuropsychological Test Battery (Unspecified Tests) | Battery |  | 3 |
| NIH Dimensional Card Sort | Executive function | NIH Toolbox | 1 |
| NIH Flanker Inhibitory Control | Executive function | NIH Toolbox | 1 |
| Numbers Test (Unspecified Test) | Attention |  | 1 |
| Oktem Verbal Learning Processes - Learning, Recall and Recognition | Memory |  | 1 |
| Orientation test (Unspecified Test) | Orientation |  | 1 |
| Pentagon Copy (Timed, Non-standardised Test) | Psychomotor speed |  | 3 |
| Letter Fluency - FAS (English) | Executive function / Verbal fluency |  | 5 |
| Letter Fluency - FAS (Spanish / Catalan) | Executive function / Verbal fluency |  | 2 |
| Letter Fluency - FPL (Italian) | Executive function / Verbal fluency |  | 1 |
| Letter Fluency - Fu, A, Ni (Japanese) | Executive function / Verbal fluency |  | 1 |
| Letter Fluency - KAS (Turkish) | Executive function / Verbal fluency |  | 1 |
| Letter Fluency - NKP (Czech) | Executive function / Verbal fluency |  | 2 |
| Letter Fluency - P (Spanish) | Executive function / Verbal fluency |  | 1 |
| **Supplementary Table 2.** (Continued) | | | |
| **Cognitive Test** | **Cognitive Domain /**  **Test Type** | **Battery** | **Studies (n)** |
| Letter Fluency - PAS (Finnish) | Executive function / Verbal fluency |  | 1 |
| Letter Fluency - S (Spanish / Catalan) | Executive function / Verbal fluency |  | 1 |
| Letter Fluency - XƩA (Greek) | Executive function / Verbal fluency |  | 1 |
| Letter Fluency - Unspecified Letter(s) | Executive function / Verbal fluency |  | 4 |
| Phrase construction (Unspecified Test) | Language |  | 1 |
| Proverbs (Unspecified Test) | Executive function |  | 1 |
| Purdue Pegboard Test | Psychomotor speed |  | 4 |
| Raven's Coloured Progressive Matrices | Nonverbal IQ test |  | 3 |
| Reaction Time - Continuous | Reaction time |  | 1 |
| Reaction Time - Simple | Reaction time |  | 1 |
| Reaction Time - Target | Reaction time |  | 1 |
| Reaction Time (Unspecified Test) | Reaction time |  | 2 |
| Repeatable Battery for the Assessment of Neuropsychological Status (RBANS) Coding | Processing speed | RBANS | 1 |
| RBANS Digit Span | Attention | RBANS | 1 |
| RBANS Figure Copy | Visuospatial function | RBANS | 1 |
| RBANS Figure Recall | Memory | RBANS | 1 |
| RBANS Line Orientation | Visuospatial function | RBANS | 1 |
| RBANS List Learning | Memory | RBANS | 1 |
| RBANS Naming | Language | RBANS | 1 |
| RBANS Semantic Fluency | Language / Verbal fluency | RBANS | 1 |
| **Supplementary Table 2.** (Continued) | | | |
| **Cognitive Test** | **Cognitive Domain /**  **Test Type** | **Battery** | **Studies (n)** |
| RBANS Story Memory | Memory | RBANS | 1 |
| Rey Auditory Verbal Learning Test (RAVLT) - Learning, Recall and Recognition | Memory |  | 32 |
| Rey Complex Figure - Copy | Visuospatial function |  | 11 |
| Rey Complex Figure - 3-minute Recall and Delayed Recall | Memory |  | 11 |
| Rivermead Behavioural Memory Test (RBMT) Story Immediate and Delayed Recall | Memory |  | 3 |
| RBMT Picture Recognition | Memory |  | 1 |
| Semantic Fluency - Animals (Czech) | Language / Verbal fluency |  | 1 |
| Semantic Fluency - Animals (English) | Language / Verbal fluency |  | 1 |
| Semantic Fluency - Animals (Japanese) | Language / Verbal fluency |  | 1 |
| Semantic Fluency - Animals (Spanish / Catalan) | Language / Verbal fluency |  | 5 |
| Semantic Fluency - Animals (Spanish) | Language / Verbal fluency |  | 1 |
| Semantic Fluency - Animals (Turkish) | Language / Verbal fluency |  | 1 |
| Semantic Fluency - Vegetables | Language / Verbal fluency |  | 1 |
| Semantic Fluency - Unspecified Category | Language / Verbal fluency |  | 8 |
| Sentence Repetition | Language / Working memory |  | 1 |
| Serial Dotting Test | Psychomotor speed |  | 3 |
| Spatial Span (Unspecified Test) | Working memory |  | 1 |
| Stroop Colour Naming | Processing speed |  | 21 |
| Stroop Colour Word (Interference) Test | Executive function |  | 30 |
| Symbol Digit Modalities Test (SDMT) | Processing speed |  | 5 |
| **Supplementary Table 2.** (Continued) | | | |
| **Cognitive Test** | **Cognitive Domain /**  **Test Type** | **Battery** | **Studies (n)** |
| Tactile Verbal Memory (Unspecified Test) | Memory |  | 1 |
| Token Test | Language |  | 2 |
| Tracks Task | Psychomotor speed |  | 2 |
| Trail Making Test A (TMT-A) | Processing speed / Psychomotor speed |  | 37 |
| Trail Making Test B (TMT-B) | Executive function |  | 24 |
| Verbal Analogies Test | Executive function |  | 1 |
| Visual Discrimination Test - Complex Form | Visuospatial function |  | 1 |
| Visual Discrimination Test - Directional Discrimination | Visuospatial function |  | 1 |
| Visual Discrimination Test - Length and Size | Visuospatial function |  | 1 |
| Visual Discrimination Test - Overlapping Figures | Visuospatial function |  | 1 |
| Visual Discrimination Test - Visual Counting | Visuospatial function |  | 1 |
| Visual Gestalts - Learning and Retention | Memory |  | 1 |
| Visual Memory - Recall (Unspecified Test) | Memory |  | 2 |
| Visual Memory - Recognition (Unspecified Test) | Memory |  | 1 |
| Wechsler Adult Intelligence Scale (WAIS) Full Scale IQ | IQ test | WAIS | 3 |
| WAIS Block Design | Executive function / Visuospatial function | WAIS | 6 |
| WAIS Coding / Digit Symbol | Processing speed | WAIS | 6 |
| WAIS Comprehension | Language | WAIS | 1 |
| WAIS Digit Span (Total) | Attention | WAIS | 1 |
| WAIS Digit Span Backward | Attention / Working memory | WAIS | 12 |
| **Supplementary Table 2.** (Continued) | | | |
| **Cognitive Test** | **Cognitive Domain /**  **Test Type** | **Battery** | **Studies (n)** |
| WAIS Digit Span Forward | Attention | WAIS | 11 |
| WAIS Similarities | Language / Executive function | WAIS | 1 |
| WAIS Symbol Search | Processing speed | WAIS | 3 |
| WAIS Vocabulary | Language | WAIS | 1 |
| Wechsler Memory Scale (WMS) Designs - Immediate and Delayed Recall | Memory | WMS | 1 |
| WMS Digit Span Backward | Attention / Working memory | WMS | 13 |
| WMS Digit Span Forward | Attention | WMS | 13 |
| WMS Information and Orientation | Orientation | WMS | 5 |
| WMS Logical Memory - Immediate and Delayed Recall | Memory | WMS | 10 |
| WMS Mental Control | Attention / Working memory | WMS | 8 |
| WMS Paired Associates - Learning and Recall | Memory | WMS | 5 |
| WMS Visual Reproduction - Immediate and Delayed Recall | Memory | WMS | 8 |
| Western Aphasia Battery Object Naming | Language |  | 1 |
| Word List Immediate Recall, Delayed Recall and Recognition (Unspecified Test) | Memory |  | 4 |
| **Note.** Tests with multiple editions or alternate forms have been grouped under a unified heading, where applicable (e.g. WAIS, WMS); Unspecified Test = study did not provide the exact test name or version; The listed cognitive domain reflects either the primary domain classified in the source article or the most commonly accepted classification. | | | |

| **Supplementary Table 3.** Summary of sensitivity analyses for cognitive outcomes following shunt surgery in iNPH | | | | | | | |  |
| --- | --- | --- | --- | --- | --- | --- | --- | --- |
| **Outcome** | **Studies**  **(*k*)** | **After exclusion**  **(*k*)** | **Original**  **MD**  **[95% CI]** | **After exclusion MD**  **[95% CI]** | **I² change (%)** | **Trim-and-Fill**  **adjusted MD**  **[95% CI]** | **Interpretation** | |
| DSB | 10 | 8 | 0.33  [0.10, 0.55] | 0.30  [0.07, 0.52] | 64.3 to 65.6 | 0.20  [–0.01, 0.42] | Outlier exclusion slightly reduced effect magnitude, with direction and significance preserved. | |
| DSF | 10 | 9 | 0.10  [–0.03, 0.24] | 0.11  [–0.03, 0.24] | 9.40 to 11.3 | 0.02  [–0.12, 0.15] | No significant effect detected; results stable after excluding high-variance study. | |
| FAB | 24 | 22 | 1.56  [1.04, 2.08] | 1.25  [0.88, 1.63] | 60.0 to 17.1 | 1.02  [0.62, 1.42] | Effect robust; exclusion reduced heterogeneity without altering effect direction or significance. | |
| GPB-D | 5 | 4 | –31.54  [–43.47, –19.61] | –25.60  [–38.36, –12.84] | 10.7 to 0.0 | –24.50  [–36.63, –12.37] | Effect direction preserved with slight attenuation; formal sensitivity limited (*k* < 10). | |
| MoCA | 7 | 5 | 1.75  [0.79, 2.71] | 1.47  [0.48, 2.47] | 22.4 to 27.4 | 1.47  [0.48, 2.47] | Slight reduction in effect magnitude; low heterogeneity and significance preserved. | |
| MMSE | 71 | 66 | 1.57  [1.23, 1.91] | 1.44  [1.15, 1.73] | 61.0 to 45.4 | 1.12  [0.80, 1.44] | Effect robust; magnitude attenuated after exclusion, with reduced heterogeneity and preserved significance. | |
| RAVLT-Learning | 12 | 9 | 5.58  [3.77, 7.39] | 5.07  [3.26, 6.88] | 41.3 to 44.2 | 4.41  [2.66, 6.15] | Stable after excluding small studies; no material change in effect size or heterogeneity. | |
| RAVLT-  Delayed Recall | 11 | 9 | 1.37  [0.86, 1.88] | 1.38  [0.85, 1.92] | 42.9 to 50.0 | 1.04  [0.54, 1.54] | Stable after excluding small studies; no material change in effect size or heterogeneity. | |
| TMT-A | 21 | 20 | –22.73  [–31.35, –14.11] | –24.17  [–33.90, –14.44] | 16.6 to 20.7 | –14.72  [–25.37, –4.07] | Effect robust; minor heterogeneity change after exclusion, with significance preserved. | |
| TMT-B (outlier exclusion) | 13 | 12 | –35.18  [–59.91, –10.46] | –39.72  [–64.14, –15.30] | 47.9 to 44.9 | –35.98  [–61.03, –10.93] | Effect robust; exclusion slightly increased improvement with no meaningful change in heterogeneity. | |
| TMT-B  (small study exclusion) | 13 | 10 | –35.18  [–59.91, –10.46] | –28.59  [–52.61, –4.57] | 47.9 to 48.3 | No imputed  studies | Effect robust; mild decrease in effect magnitude with no meaningful change in heterogeneity. | |
| **Note.** MD = Mean difference. I² change indicates pre- to post-exclusion heterogeneity. Sensitivity analyses were limited when *k* < 10 and are therefore interpreted descriptively rather than as formal robustness tests. Trim-and-fill results are shown for completeness but are underpowered in small meta-analyses. Exclusions targeted the most influential or high-variance studies for each outcome (see Methods). | | | | | | | | |

**Supplementary Text S3. Sensitivity analyses interpretive summary**

Sensitivity analyses summarised in Supplementary Table 3 confirmed the robustness of pooled cognitive effects across all outcomes. Stepwise exclusion of small, high-variance, or directionally inconsistent studies did not materially alter the direction or significance of any pooled estimates. For key outcomes, TMT-A and TMT-B remained significant after exclusion of influential datasets, indicating consistent improvements in processing speed/psychomotor speed and executive function. In TMT-B, removal of an outlier dataset (Poca et al., 2004) increased the magnitude of the pooled effect (-35.18s to -39.72s) without altering heterogeneity. For GPB-D, exclusion of the largest study (Hellstrom et al., 2012) modestly reduced the pooled mean difference (–31.5s to –25.6s) but did not alter its direction or significance, supporting robustness despite the small number of studies. Across all remaining measures (DSB, DSF, FAB, MoCA, MMSE, RAVLT Learning, RAVLT Delayed Recall), exclusion of high-influence or outlier studies minimally affected pooled estimates and slightly reduced heterogeneity. Trim-and-fill analyses indicated limited funnel-plot asymmetry and minor attenuation of pooled effects, supporting the overall stability of the findings.

**Supplementary Figure 1.** Digit Span Backward (DSB)

**Note.** The forest plot illustrates the difference in DSB scores post-shunt surgery. The pooled mean difference was calculated using a random-effects model. Individual study estimates are presented with their 95% confidence intervals. Average differences are also shown with 95% confidence intervals. Unit of measurement = Maximum span length (number of digits correctly recalled in reverse order), based on standard Wechsler administration and scoring procedures.

**Supplementary Figure 2.** Digit Span Forward (DSF)

**Note.** The forest plot illustrates the difference in DSF scores post-shunt surgery. The pooled mean difference was calculated using a random-effects model. Individual study estimates are presented with their 95% confidence intervals. Average differences are also shown with 95% confidence intervals. Unit of measurement = Maximum span length (number of digits correctly recalled in forward order), based on standard Wechsler administration and scoring procedures.

**Supplementary Figure 3.** Frontal Assessment Battery (FAB)

**Note.** The forest plot illustrates the difference in FAB scores post-shunt surgery. The pooled mean difference was calculated using a random-effects model. Individual study estimates are presented with their 95% confidence intervals. Average differences are also shown with 95% confidence intervals. Unit of measurement = Raw score based on a maximum of 18.

**Supplementary Figure 4.** Rey Auditory Verbal Learning Test (RAVLT) Learning

**Note.** The forest plot illustrates the difference in RAVLT Learning Trials scores post-shunt surgery. The pooled mean difference was calculated using a random-effects model. Individual study estimates are presented with their 95% confidence intervals. Average differences are also shown with 95% confidence intervals. Unit of measurement = Total number of words recalled across the learning trials.

**Supplementary Figure 5.** Rey Auditory Verbal Learning Test (RAVLT) Delayed Recall

**Note.** The forest plot illustrates the difference in RAVLT Delayed Recall scores post-shunt surgery. The pooled mean difference was calculated using a random-effects model. Individual study estimates are presented with their 95% confidence intervals. Average differences are also shown with 95% confidence intervals. Unit of measurement = Total number of words recalled following a delay, based on a maximum of 15.

**Supplementary Figure 6.** Montreal Cognitive Assessment (MoCA)

**Note.** The forest plot illustrates the difference in MoCA scores post-shunt surgery. The pooled mean difference was calculated using a random-effects model. Individual study estimates are presented with their 95% confidence intervals. Average differences are also shown with 95% confidence intervals. Unit of measurement = Raw score based on a maximum of 30.

**Supplementary Figure 7.** Mini-Mental State Exam (MMSE)

**Note.** The forest plot illustrates the difference in MMSE scores post-shunt surgery. The pooled mean difference was calculated using a random-effects model. Individual study estimates are presented with their 95% confidence intervals. Average differences are also shown with 95% confidence intervals. Unit of measurement = Raw score based on a maximum of 30.

**Full Reference List of the Included Studies**

Abu Hamdeh S, Virhammar J, Sehlin D, Alafuzoff I, Cesarini KG, Marklund N. Brain tissue Aβ42 levels are linked to shunt response in idiopathic normal pressure hydrocephalus. J Neurosurg. 2019 Jan 1;130(1):121-129.

Acosta LMY, Stubblefield K, Conwell T, Espaillat K, Koons H, Konrad P, Fang J, Kirshner H, Davis T. Protocolizing the Workup for Idiopathic Normal Pressure Hydrocephalus Improves Outcomes. Neurol Clin Pract. 2021 Aug;11(4):e447-e453.

Agerskov S, Hellström P, Andrén K, Kollén L, Wikkelsö C, Tullberg M. The phenotype of idiopathic normal pressure hydrocephalus-a single center study of 429 patients. J Neurol Sci. 2018 Aug 15;391:54-60.

Akiba C, Nakajima M, Miyajima M, Ogino I, Motoi Y, Kawamura K, Adachi S, Kondo A, Sugano H, Tokuda T, Irie K, Arai H. Change of Amyloid-β 1-42 Toxic Conformer Ratio After Cerebrospinal Fluid Diversion Predicts Long-Term Cognitive Outcome in Patients with Idiopathic Normal Pressure Hydrocephalus. J Alzheimers Dis. 2018;63(3):989-1002.

Akiguchi I, Ishii M, Watanabe Y, Watanabe T, Kawasaki T, Yagi H, Shiino A, Shirakashi Y, Kawamoto Y. Shunt-responsive parkinsonism and reversible white matter lesions in patients with idiopathic NPH. J Neurol. 2008 Sep;255(9):1392-9.

Andrén K, Wikkelsø C, Tisell M, Hellström P. Natural course of idiopathic normal pressure hydrocephalus. J Neurol Neurosurg Psychiatry. 2014 Jul;85(7):806-10.

Andrén K, Wikkelsø C, Laurell K, Kollén L, Hellström P, Tullberg M. Symptoms and signs did not predict outcome after surgery: a prospective study of 143 patients with idiopathic normal pressure hydrocephalus. J Neurol. 2024 Jun;271(6):3215-3226.

Asahara Y, Suda M, Omoto S, Kobayashi K, Atsuchi M, Nagashima H, Suzuki M. Predictive Ability of Frontal Assessment Battery for Cognitive Improvement After Shunt Surgery in Individuals With Idiopathic Normal Pressure Hydrocephalus. Cogn Behav Neurol. 2023 Dec 1;36(4):228-236.

Aygok G, Marmarou A, Young HF. Three-year outcome of shunted idiopathic NPH patients. Acta Neurochir Suppl. 2005;95:241-5.

Behrens A, Elgh E, Leijon G, Kristensen B, Eklund A, Malm J. The Computerized General Neuropsychological INPH Test revealed improvement in idiopathic normal pressure hydrocephalus after shunt surgery. J Neurosurg. 2019 Feb 8;132(3):733-740.

Belotti F, Pertichetti M, Muratori A, Migliorati K, Panciani PP, Draghi R, Godano U, Borghesi I, Fontanella MM. Idiopathic normal pressure hydrocephalus: postoperative patient perspective and quality of life. Acta Neurochir (Wien). 2022 Nov;164(11):2855-2866.

Bloch O, McDermott MW. Lumboperitoneal shunts for the treatment of normal pressure hydrocephalus. J Clin Neurosci. 2012 Aug;19(8):1107-11.

Broggi M, Redaelli V, Tringali G, Restelli F, Romito L, Schiavolin S, Tagliavini F, Broggi G. Normal Pressure Hydrocephalus and Parkinsonism: Preliminary Data on Neurosurgical and Neurological Treatment. World Neurosurg. 2016 Jun;90:348-356.

Bugalho P, Alves L, Ribeiro O. Normal pressure hydrocephalus: a qualitative study on outcome. Arq Neuropsiquiatr. 2013 Nov;71(11):890-5.

Bubeníková A, Sedlák V, Skalický P, Rýdlo O, Haratek K, Vlasák A, Leško R, Netuka D, Beneš V 3rd, Beneš V, Bradáč O. Clinical Improvement after Shunt Surgery in Patients with Idiopathic Normal Pressure Hydrocephalus Can Be Quantified by Diffusion Tensor Imaging. AJNR Am J Neuroradiol. 2025 Apr 2;46(4):766-773.

Büyükgök D, Özdemir Ö, Ünal TC, Barlas O. When to Assess: Cognitive Impact of Ventriculoperitoneal Shunt Operation in Elderly Adults with Normal Pressure Hydrocephalus. World Neurosurg. 2021 Oct;154:e302-e312.

Cage TA, Auguste KI, Wrensch M, Wu YW, Gupta N. Self-reported functional outcome after surgical intervention in patients with idiopathic normal pressure hydrocephalus. J Clin Neurosci. 2011 May;18(5):649-54.

Calcagni ML, Lavalle M, Mangiola A, Indovina L, Leccisotti L, De Bonis P, Marra C, Pelliccioni A, Anile C, Giordano A. Early evaluation of cerebral metabolic rate of glucose (CMRglu) with 18F-FDG PET/CT and clinical assessment in idiopathic normal pressure hydrocephalus (INPH) patients before and after ventricular shunt placement: preliminary experience. Eur J Nucl Med Mol Imaging. 2012 Feb;39(2):236-41.

Caruso JP, El Ahmadieh TY, Trent T, Stutzman SE, Anderson R, Schneider N, Woodruff C, Adenwalla A, Wang J, Almekkawi AK, Venkatachalam A, Olson DM, Aoun SG, White JA. Neurologic Quality of Life Outcomes in Patients with Normal Pressure Hydrocephalus After Ventriculoperitoneal Shunt Placement: A Prospective Assessment of Cognition, Mobility, and Social Participation. World Neurosurg. 2024 Oct;190:e26-e33.

Chang S, Agarwal S, Williams MA, Rigamonti D, Hillis AE. Demographic factors influence cognitive recovery after shunt for normal-pressure hydrocephalus. Neurologist. 2006 Jan;12(1):39-42.

Chaudhry P, Kharkar S, Heidler-Gary J, Hillis AE, Newhart M, Kleinman JT, Davis C, Rigamonti D, Wang P, Irani DN, Williams MA. Characteristics and reversibility of dementia in Normal Pressure Hydrocephalus. Behav Neurol. 2007;18(3):149-58.

Chen IH, Huang CI, Liu HC, Chen KK. Effectiveness of shunting in patients with normal pressure hydrocephalus predicted by temporary, controlled-resistance, continuous lumbar drainage: a pilot study. J Neurol Neurosurg Psychiatry. 1994 Nov;57(11):1430-2.

Chen J, He W, Zhang X, Lv M, Zhou X, Yang X, Wei H, Ma H, Li H, Xia J. Value of MRI-based semi-quantitative structural neuroimaging in predicting the prognosis of patients with idiopathic normal pressure hydrocephalus after shunt surgery. Eur Radiol. 2022 Nov;32(11):7800-7810.

Chen J, Xian J, Wang F, Zuo C, We L, Chen Z, Hu R, Feng H. Long-term outcomes of ventriculoperitoneal shunt therapy in idiopathic normal pressure hydrocephalus. BMC Surg. 2025 Apr 12;25(1):157.

Chiaravalloti A, Filippi L, Bagni O, Schillaci O, Czosnyka Z, Czosnyka M, de Pandis MF, Federici G, Galli M, Pompucci A, Petrella G. Cortical metabolic changes and clinical outcome in normal pressure hydrocephalus after ventriculoperitoneal shunt: Our preliminary results. Rev Esp Med Nucl Imagen Mol (Engl Ed). 2020 Nov-Dec;39(6):367-374.

Chidiac C, Sundström N, Tullberg M, Arvidsson L, Olivecrona M. Waiting time for surgery influences the outcome in idiopathic normal pressure hydrocephalus - a population-based study. Acta Neurochir (Wien). 2022 Feb;164(2):469-478.

Craven CL, Toma AK, Mostafa T, Patel N, Watkins LD. The predictive value of DESH for shunt responsiveness in idiopathic normal pressure hydrocephalus. J Clin Neurosci. 2016 Dec;34:294-298.

Damasceno BP, Carelli EF, Honorato DC, Facure JJ. The predictive value of cerebrospinal fluid tap-test in normal pressure hydrocephalus. Arq Neuropsiquiatr. 1997 Jun;55(2):179-85.

de Oliveira MF, Sorte AAB Jr, Emerenciano DL, Rotta JM, Mendes GAS, Pinto FCG. Long term follow-up of shunted idiopathic normal pressure hydrocephalus patients: a single center experience. Acta Neurol Belg. 2021 Dec;121(6):1799-1806.

del Mar Matarín M, Pueyo R, Poca MA, Falcón C, Mataró M, Bargalló N, Sahuquillo J, Junqué C. Post-surgical changes in brain metabolism detected by magnetic resonance spectroscopy in normal pressure hydrocephalus: results of a pilot study. J Neurol Neurosurg Psychiatry. 2007 Jul;78(7):760-3.

Di Rienzo A, Carrassi E, Dobran M, Colasanti R, Capece M, Aiudi D, Iacoangeli M. Ventriculoatrial Shunting: An Escape Option in Patients with Idiopathic Normal Pressure Hydrocephalus Failing Ventriculoperitoneal Drainage. World Neurosurg. 2022 Jan;157:e286-e293.

Dixon GR, Friedman JA, Luetmer PH, Quast LM, McClelland RL, Petersen RC, Maher CO, Ebersold MJ. Use of cerebrospinal fluid flow rates measured by phase-contrast MR to predict outcome of ventriculoperitoneal shunting for idiopathic normal-pressure hydrocephalus. Mayo Clin Proc. 2002 Jun;77(6):509-14.

Duinkerke A, Williams MA, Rigamonti D, Hillis AE. Cognitive recovery in idiopathic normal pressure hydrocephalus after shunt. Cogn Behav Neurol. 2004 Sep;17(3):179-84.

Eleftheriou A, Blystad I, Tisell A, Gasslander J, Lundin F. Publisher Correction: Indication of Thalamo-Cortical Circuit Dysfunction in Idiopathic Normal Pressure Hydrocephalus: A Diffusion Tensor Imaging Study. Sci Rep. 2020 Jul 16;10(1):12014. Erratum for: Sci Rep. 2020 Apr 9;10(1):6148.

Eleftheriou A, Ulander M, Lundin F. Circadian rhythm in idiopathic normal pressure hydrocephalus. Clin Neurol Neurosurg. 2018 Jan;164:72-74.

Fang X, Deng Y, Xu X, Shu W, Tang F, Li S, Zhu T, Zhang L, Zhong P, Mao R. One-year outcome of a lumboperitoneal shunt in older adults with idiopathic normal pressure hydrocephalus. Front Surg. 2022 Sep 21;9:977123.

Farahmand D, Sæhle T, Eide PK, Tisell M, Hellström P, Wikkelsö C. A double-blind randomized trial on the clinical effect of different shunt valve settings in idiopathic normal pressure hydrocephalus. J Neurosurg. 2016 Feb;124(2):359-67.

Foss T, Eide PK, Finset A. Intracranial pressure parameters in idiopathic normal pressure hydrocephalus patients with or without improvement of cognitive function after shunt treatment. Dement Geriatr Cogn Disord. 2007;23(1):47-54.

Gago MF, Ferreira F, Bicho E. Quantitative gait analysis value as a predictor of shunt surgery effectiveness in normal pressure hydrocephalus: A technical note. Clin Neurol Neurosurg. 2022 Oct;221:107405.

Giannini G, Palandri G, Ferrari A, Oppi F, Milletti D, Albini-Riccioli L, Mantovani P, Magnoni S, Chiari L, Cortelli P, Cevoli S; BOLOGNA PRO-HYDRO Study Group. A prospective evaluation of clinical and instrumental features before and after ventriculo-peritoneal shunt in patients with idiopathic Normal pressure hydrocephalus: The Bologna PRO-Hydro study. Parkinsonism Relat Disord. 2019 Sep;66:117-124.

Gleichgerrcht E, Cervio A, Salvat J, Loffredo AR, Vita L, Roca M, Torralva T, Manes F. Executive function improvement in normal pressure hydrocephalus following shunt surgery. Behav Neurol. 2009;21(3):181-5.

Goertz L, Pieczewski J, Zopfs D, Kabbasch C, Timmer M, Goldbrunner R, Wetzel C. Prospective evaluation of flow-regulated valves for idiopathic normal pressure hydrocephalus: 1-year results. J Clin Neurosci. 2024 Jun;124:94-101.

Gold D, Wisialowski C, Piryatinsky I, Malloy P, Correia S, Salloway S, Klinge P, Gaudet CE, Niermeyer M, Lee A. Longitudinal post-shunt outcomes in idiopathic normal pressure hydrocephalus with and without comorbid Alzheimer's disease. J Int Neuropsychol Soc. 2023 Oct;29(8):751-762.

Golomb J, Wisoff J, Miller DC, Boksay I, Kluger A, Weiner H, Salton J, Graves W. Alzheimer's disease comorbidity in normal pressure hydrocephalus: prevalence and shunt response. J Neurol Neurosurg Psychiatry. 2000 Jun;68(6):778-81.

Graff-Radford NR, Godersky JC. Normal-pressure hydrocephalus. Onset of gait abnormality before dementia predicts good surgical outcome. Arch Neurol. 1986 Sep;43(9):940-2.

Grasso G, Torregrossa F, Leone L, Frisella A, Landi A. Long-Term Efficacy of Shunt Therapy in Idiopathic Normal Pressure Hydrocephalus. World Neurosurg. 2019 Sep;129:e458-e463.

Grasso G, Torregrossa F. The impact of cerebrospinal fluid shunting on quality of life in idiopathic normal pressure hydrocephalus: a long-term analysis. Neurosurg Focus. 2023 Apr;54(4):E7.

Hallqvist C, Grönstedt H, Arvidsson L. Gait, falls, cognitive function, and health-related quality of life after shunt-treated idiopathic normal pressure hydrocephalus-a single-center study. Acta Neurochir (Wien). 2022 Sep;164(9):2367-2373.

Hamilton R, Patel S, Lee EB, Jackson EM, Lopinto J, Arnold SE, Clark CM, Basil A, Shaw LM, Xie SX, Grady MS, Trojanowski JQ. Lack of shunt response in suspected idiopathic normal pressure hydrocephalus with Alzheimer disease pathology. Ann Neurol. 2010 Oct;68(4):535-40.

Hasselbalch SG, Carlsen JF, Alaouie MM, Munch TN, Holst AV, Taudorf S, Rørvig-Løppentien C, Juhler M, Waldemar G. Prediction of shunt response in idiopathic normal pressure hydrocephalus by combined lumbar infusion test and preoperative imaging scoring. Eur J Neurol. 2023 Oct;30(10):3047-3055.

Hashimoto M, Ishikawa M, Mori E, Kuwana N; Study of INPH on neurological improvement (SINPHONI). Diagnosis of idiopathic normal pressure hydrocephalus is supported by MRI-based scheme: a prospective cohort study. Cerebrospinal Fluid Res. 2010 Oct 31;7:18.

Hellström P, Klinge P, Tans J, Wikkelsø C. The neuropsychology of iNPH: findings and evaluation of tests in the European multicentre study. Clin Neurol Neurosurg. 2012 Feb;114(2):130-4.

Hellström P, Edsbagge M, Blomsterwall E, Archer T, Tisell M, Tullberg M, Wikkelsø C. Neuropsychological effects of shunt treatment in idiopathic normal pressure hydrocephalus. Neurosurgery. 2008 Sep;63(3):527-35; discussion 535-6.

He WJ, Zhang XJ, Xu QZ, Bai RT, Chen JK, Zhou X, Xia J. Are preoperative phase-contrast CSF flow parameters ideal for predicting the outcome of shunt surgery in patients with idiopathic normal pressure hydrocephalus? Front Neurol. 2022 Sep 27;13:959450.

Hiraoka K, Yamasaki H, Takagi M, Saito M, Nishio Y, Iizuka O, Kanno S, Kikuchi H, Kondo T, Mori E. Changes in the volumes of the brain and cerebrospinal fluid spaces after shunt surgery in idiopathic normal-pressure hydrocephalus. J Neurol Sci. 2010 Sep 15;296(1-2):7-12.

Hiraoka K, Narita W, Kikuchi H, Baba T, Kanno S, Iizuka O, Tashiro M, Furumoto S, Okamura N, Furukawa K, Arai H, Iwata R, Mori E, Yanai K. Amyloid deposits and response to shunt surgery in idiopathic normal-pressure hydrocephalus. J Neurol Sci. 2015 Sep 15;356(1-2):124-8.

Hong YJ, Kim MJ, Jeong E, Kim JE, Hwang J, Lee JI, Lee JH, Na DL. Preoperative biomarkers in patients with idiopathic normal pressure hydrocephalus showing a favorable shunt surgery outcome. J Neurol Sci. 2018 Apr 15;387:21-26.

Huang W, Fang X, Li S, Mao R, Ye C, Liu W, Deng Y, Lin G. Abnormal characteristic static and dynamic functional network connectivity in idiopathic normal pressure hydrocephalus. CNS Neurosci Ther. 2024 Mar;30(3):e14178.

Huang W, Fang X, Li S, Mao R, Ye C, Liu W, Lin G. Shunt Surgery Efficacy Is Correlated With Baseline Cerebrum Perfusion in Idiopathic Normal Pressure Hydrocephalus: A 3D Pulsed Arterial-Spin Labeling Study. Front Aging Neurosci. 2022 Feb 23;14:797803.

Hülser M, Spielmann H, Oertel J, Sippl C. Motor skills, cognitive impairment, and quality of life in normal pressure hydrocephalus: early effects of shunt placement. Acta Neurochir (Wien). 2022 Jul;164(7):1765-1775.

Iddon JL, Pickard JD, Cross JJ, Griffiths PD, Czosnyka M, Sahakian BJ. Specific patterns of cognitive impairment in patients with idiopathic normal pressure hydrocephalus and Alzheimer's disease: a pilot study. J Neurol Neurosurg Psychiatry. 1999 Dec;67(6):723-32.

Illán-Gala I, Pérez-Lucas J, Martín-Montes A, Máñez-Miró J, Arpa J, Ruiz-Ares G. Long-term outcomes of adult chronic idiopathic hydrocephalus treated with a ventriculo-peritoneal shunt. Neurologia (Engl Ed). 2017 May;32(4):205-212.

Ishikawa M, Yamada S, Miyajima M, Kazui H, Mori E. Improvement in the long-term care burden after surgical treatment of patients with idiopathic normal pressure hydrocephalus: a supplementary study. Sci Rep. 2021 Jun 3;11(1):11732.

Ishikawa M, Mori E. Association of gait and cognition after surgery in patients with idiopathic normal pressure hydrocephalus. Sci Rep. 2023 Oct 27;13(1):18460.

Jingami N, Uemura K, Asada-Utsugi M, Kuzuya A, Yamada S, Ishikawa M, Kawahara T, Iwasaki T, Atsuchi M, Takahashi R, Kinoshita A. Two-Point Dynamic Observation of Alzheimer's Disease Cerebrospinal Fluid Biomarkers in Idiopathic Normal Pressure Hydrocephalus. J Alzheimers Dis. 2019;72(1):271-277.

Junkkari A, Häyrinen A, Rauramaa T, Sintonen H, Nerg O, Koivisto AM, Roine RP, Viinamäki H, Soininen H, Luikku A, Jääskeläinen JE, Leinonen V. Health-related quality-of-life outcome in patients with idiopathic normal-pressure hydrocephalus - a 1-year follow-up study. Eur J Neurol. 2017 Jan;24(1):58-66.

Kajimoto Y, Kameda M, Kambara A, Kuroda K, Tsuji S, Nikaido Y, Saura R, Wanibuchi M. Impact of Early Intervention for Idiopathic Normal Pressure Hydrocephalus on Long-Term Prognosis in Prodromal Phase. Front Neurol. 2022 Apr 11;13:866352.

Kambara A, Kajimoto Y, Yagi R, Ikeda N, Furuse M, Nonoguchi N, Kawabata S, Kuroiwa T, Kuroda K, Tsuji S, Saura R, Wanibuchi M. Long-Term Prognosis of Cognitive Function in Patients With Idiopathic Normal Pressure Hydrocephalus After Shunt Surgery. Front Aging Neurosci. 2021 Jan 20;12:617150.

Kamohara C, Nakajima M, Kawamura K, Akiba C, Ogino I, Xu H, Karagiozov K, Arai H, Miyajima M. Neuropsychological tests are useful for predicting comorbidities of idiopathic normal pressure hydrocephalus. Acta Neurol Scand. 2020 Dec;142(6):623-631.

Kanemoto H, Kazui H, Suzuki Y, Sato S, Kishima H, Yoshimine T, Yoshiyama K. Effect of lumbo-peritoneal shunt surgery on neuropsychiatric symptoms in patients with idiopathic normal pressure hydrocephalus. J Neurol Sci. 2016 Feb 15;361:206-12.

Kanemoto H, Kazui H, Suehiro T, Kishima H, Suzuki Y, Sato S, Azuma S, Matsumoto T, Yoshiyama K, Shimosegawa E, Tanaka T, Ikeda M. Apathy and right caudate perfusion in idiopathic normal pressure hydrocephalus: A case-control study. Int J Geriatr Psychiatry. 2019 Mar;34(3):453-462.

Kanno S, Ogawa KI, Kikuchi H, Toyoshima M, Abe N, Sato K, Miyazawa K, Oshima R, Ohtomo S, Arai H, Shibuya S, Suzuki K. Reduced default mode network connectivity relative to white matter integrity is associated with poor cognitive outcomes in patients with idiopathic normal pressure hydrocephalus. BMC Neurol. 2021 Sep 13;21(1):353.

Kanno S, Saito M, Kashinoura T, Nishio Y, Iizuka O, Kikuchi H, Takagi M, Iwasaki M, Takahashi S, Mori E. A change in brain white matter after shunt surgery in idiopathic normal pressure hydrocephalus: a tract-based spatial statistics study. Fluids Barriers CNS. 2017 Jan 30;14(1):1.

Katzen H, Ravdin LD, Assuras S, Heros R, Kaplitt M, Schwartz TH, Fink M, Levin BE, Relkin NR. Postshunt cognitive and functional improvement in idiopathic normal pressure hydrocephalus. Neurosurgery. 2011 Feb;68(2):416-9.

Kazui H, Kanemoto H, Yoshiyama K, Kishima H, Suzuki Y, Sato S, Suehiro T, Azuma S, Yoshimine T, Tanaka T. Association between high biomarker probability of Alzheimer's disease and improvement of clinical outcomes after shunt surgery in patients with idiopathic normal pressure hydrocephalus. J Neurol Sci. 2016 Oct 15;369:236-241.

Kazui H, Mori E, Ohkawa S, Okada T, Kondo T, Sakakibara R, Ueki O, Nishio Y, Ishii K, Kawaguchi T, Ishikawa M, Takeda M. Predictors of the disappearance of triad symptoms in patients with idiopathic normal pressure hydrocephalus after shunt surgery. J Neurol Sci. 2013 May 15;328(1-2):64-9.

Kazui H, Mori E, Hashimoto M, Ishikawa M, Hirono N, Takeda M. Effect of shunt operation on idiopathic normal pressure hydrocephalus patients in reducing caregiver burden: evidence from SINPHONI. Dement Geriatr Cogn Disord. 2011;31(5):363-70.

Kazui H, Miyajima M, Mori E, Ishikawa M; SINPHONI-2 Investigators. Lumboperitoneal shunt surgery for idiopathic normal pressure hydrocephalus (SINPHONI-2): an open-label randomised trial. Lancet Neurol. 2015 Jun;14(6):585-94.

Kilinc MC, Kahilogullari G, Dogan I, Alpergin BC, Terzi M, Bahadir EA, Ibis MA, Caglar YS. Changes in Callosal Angle and Evans' Index After Placing a Lumboperitoneal Shunt in Patients with Idiopathic-Normal- Pressure Hydrocephalus. Turk Neurosurg. 2022;32(2):309-314.

Kito Y, Kazui H, Kubo Y, Yoshida T, Takaya M, Wada T, Nomura K, Hashimoto M, Ohkawa S, Miyake H, Ishikawa M, Takeda M. Neuropsychiatric symptoms in patients with idiopathic normal pressure hydrocephalus. Behav Neurol. 2009;21(3):165-74.

Klassen BT, Ahlskog JE. Normal pressure hydrocephalus: how often does the diagnosis hold water? Neurology. 2011 Sep 20;77(12):1119-25.

Klinge P, Hellström P, Tans J, Wikkelsø C; European iNPH Multicentre Study Group. One-year outcome in the European multicentre study on iNPH. Acta Neurol Scand. 2012 Sep;126(3):145-53.

Korhonen VE, Remes AM, Helisalmi S, Rauramaa T, Sutela A, Vanninen R, Suhonen NM, Haapasalo A, Hiltunen M, Jääskeläinen JE, Soininen H, Koivisto AM, Leinonen V. Prevalence of C9ORF72 Expansion in a Large Series of Patients with Idiopathic Normal-Pressure Hydrocephalus. Dement Geriatr Cogn Disord. 2019;47(1-2):91-103.

Krahulik D, Vaverka M, Hrabalek L, Hampl M, Halaj M, Jablonsky J, Langova K. Ventriculoperitoneal shunt in treating of idiopathic normal pressure hydrocephalus-single-center study. Acta Neurochir (Wien). 2020 Jan;162(1):1-7.

Krauss JK, Regel JP, Vach W, Jüngling FD, Droste DW, Wakhloo AK. Flow void of cerebrospinal fluid in idiopathic normal pressure hydrocephalus of the elderly: can it predict outcome after shunting? Neurosurgery. 1997 Jan;40(1):67-73; discussion 73-4.

Krauss JK, Droste DW, Vach W, Regel JP, Orszagh M, Borremans JJ, Tietz A, Seeger W. Cerebrospinal fluid shunting in idiopathic normal-pressure hydrocephalus of the elderly: effect of periventricular and deep white matter lesions. Neurosurgery. 1996 Aug;39(2):292-9; discussion 299-300.

Lee C, Seo H, Yoon SY, Chang SH, Park SH, Hwang JH, Kang K, Kim CH, Hahm MH, Park E, Ahn JY, Park KS. Clinical significance of vitamin D in idiopathic normal pressure hydrocephalus. Acta Neurochir (Wien). 2021 Jul;163(7):1969-1977.

Liouta E, Gatzonis S, Kalamatianos T, Kalyvas A, Koutsarnakis C, Liakos F, Anagnostopoulos C, Komaitis S, Giakoumettis D, Stranjalis G. Finger tapping and verbal fluency post-tap test improvement in INPH: its value in differential diagnosis and shunt-treatment outcomes prognosis. Acta Neurochir (Wien). 2017 Dec;159(12):2301-2307.

Liu JT, Su PH. The efficacy and limitation of lumboperitoneal shunt in normal pressure hydrocephalus. Clin Neurol Neurosurg. 2020 Jun;193:105748.

Liu A, Sankey EW, Jusué-Torres I, Patel MA, Elder BD, Goodwin CR, Hoffberger J, Lu J, Rigamonti D. Clinical outcomes after ventriculoatrial shunting for idiopathic normal pressure hydrocephalus. Clin Neurol Neurosurg. 2016 Apr;143:34-8.

Luciano M, Holubkov R, Williams MA, Malm J, Nagel S, Moghekar A, Eklund A, Zwimpfer T, Katzen H, Hanley DF, Hamilton MG; PENS Co-investigators and AHCRN Site PIs. Placebo-Controlled Effectiveness of Idiopathic Normal Pressure Hydrocephalus Shunting: A Randomized Pilot Trial. Neurosurgery. 2023 Mar 1;92(3):481-489.

Luciano MG, Williams MA, Hamilton MG, Katzen HL, Dasher NA, Moghekar A, Hua J, Malm J, Eklund A, Alpert Abel N, Raslan AM. A Randomized Trial of Shunting for Idiopathic Normal-Pressure Hydrocephalus. N Engl J Med. 2025 Sep 16.

Lundin F, Ledin T, Wikkelsø C, Leijon G. Postural function in idiopathic normal pressure hydrocephalus before and after shunt surgery: a controlled study using computerized dynamic posturography (EquiTest). Clin Neurol Neurosurg. 2013 Sep;115(9):1626-31.

Lundin F, Ulander M, Svanborg E, Wikkelsø C, Leijon G. How active are patients with idiopathic normal pressure hydrocephalus and does activity improve after shunt surgery? A controlled actigraphic study. Clin Neurol Neurosurg. 2013 Feb;115(2):192-6.

Ma TS, Sharma N, Grady MS. A simplified pressure adjustment clinical pathway for programmable valves in NPH patients. Clin Neurol Neurosurg. 2017 Aug;159:83-86.

Macki M, Mahajan A, Shatz R, Air EL, Novikova M, Fakih M, Elmenini J, Kaur M, Bouchard KR, Funk BA, Schwalb JM. Prevalence of Alternative Diagnoses and Implications for Management in Idiopathic Normal Pressure Hydrocephalus Patients. Neurosurgery. 2020 Oct 15;87(5):999-1007.

Malem DN, Shand Smith JD, Toma AK, Sethi H, Kitchen ND, Watkins LD. An investigation into the clinical impacts of lowering shunt opening pressure in idiopathic normal pressure hydrocephalus: A case series. Br J Neurosurg. 2015 Feb;29(1):18-22.

Malm J, Kristensen B, Stegmayr B, Fagerlund M, Koskinen LO. Three-year survival and functional outcome of patients with idiopathic adult hydrocephalus syndrome. Neurology. 2000 Aug 22;55(4):576-8.

Malm J, Kristensen B, Fagerlund M, Koskinen LO, Ekstedt J. Cerebrospinal fluid shunt dynamics in patients with idiopathic adult hydrocephalus syndrome. J Neurol Neurosurg Psychiatry. 1995 Jun;58(6):715-23.

Malm J, Kristensen B, Karlsson T, Fagerlund M, Elfverson J, Ekstedt J. The predictive value of cerebrospinal fluid dynamic tests in patients with the idiopathic adult hydrocephalus syndrome. Arch Neurol. 1995 Aug;52(8):783-9.

Mataró M, Matarín M, Poca MA, Pueyo R, Sahuquillo J, Barrios M, Junqué C. Functional and magnetic resonance imaging correlates of corpus callosum in normal pressure hydrocephalus before and after shunting. J Neurol Neurosurg Psychiatry. 2007 Apr;78(4):395-8.

Mataró M, Poca MA, Del Mar Matarín M, Catalan R, Sahuquillo J, Galard R. CSF galanin and cognition after shunt surgery in normal pressure hydrocephalus. J Neurol Neurosurg Psychiatry. 2003 Sep;74(9):1272-7.

Matsuoka T, Fujimoto K, Kawahara M. Comparison of comfortable and maximum walking speed in the 10-meter walk test during the cerebrospinal fluid tap test in iNPH patients: A retrospective study. Clin Neurol Neurosurg. 2022 Jan;212:107049.

McGovern RA, Nelp TB, Kelly KM, Chan AK, Mazzoni P, Sheth SA, Honig LS, Teich AF, McKhann GM. Predicting Cognitive Improvement in Normal Pressure Hydrocephalus Patients Using Preoperative Neuropsychological Testing and Cerebrospinal Fluid Biomarkers. Neurosurgery. 2019 Oct 1;85(4):E662-E669.

McGrath K, Laurent D, Otero O, Hey G, Tomdio M, Sorrentino Z, Riklan J, Chowdhury MAB, Isom E, Schreffler A, Musalo M, Rahman M. An Interdisciplinary Protocol for Ventriculoperitoneal Shunt Patient Selection in Normal Pressure Hydrocephalus. World Neurosurg. 2024 Jul;187:e1-e11.

Messerer M, Blanchard M, Papadimitriou K, Vandenbulcke A, Rutz D, Beaud V, Shiban E, Bally J, Allali G, Daniel RT, Cossu G. Impact of Subjective Evaluations in Predicting Response to Ventriculoperitoneal Shunt for Idiopathic Normal Pressure Hydrocephalus. World Neurosurg. 2022 Oct;166:e741-e749.

Miyajima M, Nakajima M, Ogino I, Miyata H, Motoi Y, Arai H. Soluble amyloid precursor protein α in the cerebrospinal fluid as a diagnostic and prognostic biomarker for idiopathic normal pressure hydrocephalus. Eur J Neurol. 2013 Feb;20(2):236-42.

Miyajima M, Kazui H, Mori E, Ishikawa M; SINPHONI-2 Investigators. One-year outcome in patients with idiopathic normal-pressure hydrocephalus: comparison of lumboperitoneal shunt to ventriculoperitoneal shunt. J Neurosurg. 2016 Dec;125(6):1483-1492.

Mori K. Management of idiopathic normal-pressure hydrocephalus: a multiinstitutional study conducted in Japan. J Neurosurg. 2001 Dec;95(6):970-3.

Moriya M, Miyajima M, Nakajima M, Ogino I, Arai H. Impact of cerebrospinal fluid shunting for idiopathic normal pressure hydrocephalus on the amyloid cascade. PLoS One. 2015 Mar 30;10(3):e0119973.

Mostile G, Portaro G, Certo F, Luca A, Manna R, Terranova R, Altieri R, Nicoletti A, Barbagallo GMV, Zappia M. iNPH with parkinsonism: response to lumbar CSF drainage and ventriculoperitoneal shunting. J Neurol. 2021 Apr;268(4):1254-1265.

Murakami Y, Matsumoto Y, Hoshi K, Ito H, Fuwa TJ, Yamaguchi Y, Nakajima M, Miyajima M, Arai H, Nollet K, Kato N, Nishikata R, Kuroda N, Honda T, Sakuma J, Saito K, Hashimoto Y. Rapid increase of 'brain-type' transferrin in cerebrospinal fluid after shunt surgery for idiopathic normal pressure hydrocephalus: a prognosis marker for cognitive recovery. J Biochem. 2018 Sep 1;164(3):205-213.

Nakajima M, Miyajima M, Ogino I, Sugano H, Akiba C, Domon N, Karagiozov KL, Arai H. Use of external lumbar cerebrospinal fluid drainage and lumboperitoneal shunts with Strata NSC valves in idiopathic normal pressure hydrocephalus: a single-center experience. World Neurosurg. 2015 Mar;83(3):387-93.

Nakajima M, Miyajima M, Akiba C, Ogino I, Kawamura K, Sugano H, Hara T, Tange Y, Fusegi K, Karagiozov K, Arai H. Lumboperitoneal Shunts for the Treatment of Idiopathic Normal Pressure Hydrocephalus: A Comparison of Small-Lumen Abdominal Catheters to Gravitational Add-On Valves in a Single Center. Oper Neurosurg (Hagerstown). 2018 Dec 1;15(6):634-642.

Nakajima M, Miyajima M, Ogino I, Watanabe M, Miyata H, Karagiozov KL, Arai H, Hagiwara Y, Segawa T, Kobayashi K, Hashimoto Y. Leucine-rich α-2-glycoprotein is a marker for idiopathic normal pressure hydrocephalus. Acta Neurochir (Wien). 2011 Jun;153(6):1339-46; discussion 1346.

Nakajima M, Miyajima M, Ogino I, Akiba C, Kawamura K, Kamohara C, Fusegi K, Harada Y, Hara T, Sugano H, Tange Y, Karagiozov K, Kasuga K, Ikeuchi T, Tokuda T, Arai H. Preoperative Phosphorylated Tau Concentration in the Cerebrospinal Fluid Can Predict Cognitive Function Three Years after Shunt Surgery in Patients with Idiopathic Normal Pressure Hydrocephalus. J Alzheimers Dis. 2018;66(1):319-331.

Nakajima M, Miyajima M, Ogino I, Akiba C, Sugano H, Hara T, Fusegi K, Karagiozov K, Arai H. Cerebrospinal fluid biomarkers for prognosis of long-term cognitive treatment outcomes in patients with idiopathic normal pressure hydrocephalus. J Neurol Sci. 2015 Oct 15;357(1-2):88-95.

Nakajima M, Yamada S, Miyajima M, Kawamura K, Akiba C, Kazui H, Mori E, Ishikawa M; SINPHONI-2 Investigators. Tap Test Can Predict Cognitive Improvement in Patients With iNPH-Results From the Multicenter Prospective Studies SINPHONI-1 and -2. Front Neurol. 2021 Nov 2;12:769216.

Nakatsu D, Fukuhara T, Chaytor NS, Phatak VS, Avellino AM. Repeatable Battery for the Assessment of Neuropsychological Status (RBANS) as a Cognitive Evaluation Tool for Patients with Normal Pressure Hydrocephalus. Neurol Med Chir (Tokyo). 2016;56(2):51-61.

Nakayama T, Ouchi Y, Yoshikawa E, Sugihara G, Torizuka T, Tanaka K. Striatal D2 receptor availability after shunting in idiopathic normal pressure hydrocephalus. J Nucl Med. 2007 Dec;48(12):1981-6.

Narita W, Nishio Y, Baba T, Iizuka O, Ishihara T, Matsuda M, Iwasaki M, Tominaga T, Mori E. High-Convexity Tightness Predicts the Shunt Response in Idiopathic Normal Pressure Hydrocephalus. AJNR Am J Neuroradiol. 2016 Oct;37(10):1831-1837.

Oike R, Inoue Y, Matsuzawa K, Sorimachi T. Screening for idiopathic normal pressure hydrocephalus in the elderly after falls. Clin Neurol Neurosurg. 2021 Apr 24;205:106635.

Oliveira MF, Saad F, Reis RC, Rotta JM, Pinto FC. Programmable valve represents an efficient and safe tool in the treatment of idiopathic normal-pressure hydrocephalus patients. Arq Neuropsiquiatr. 2013 Apr;71(4):229-36.

Patel S, Lee EB, Xie SX, Law A, Jackson EM, Arnold SE, Clark CM, Shaw LM, Grady MS, Trojanowski JQ, Hamilton RH. Phosphorylated tau/amyloid beta 1-42 ratio in ventricular cerebrospinal fluid reflects outcome in idiopathic normal pressure hydrocephalus. Fluids Barriers CNS. 2012 Mar 23;9(1):7.

Petersen J, Hellström P, Wikkelsø C, Lundgren-Nilsson A. Improvement in social function and health-related quality of life after shunt surgery for idiopathic normal-pressure hydrocephalus. J Neurosurg. 2014 Oct;121(4):776-84.

Peterson KA, Housden CR, Killikelly C, DeVito EE, Keong NC, Savulich G, Czosnyka Z, Pickard JD, Sahakian BJ. Apathy, ventriculomegaly and neurocognitive improvement following shunt surgery in normal pressure hydrocephalus. Br J Neurosurg. 2016;30(1):38-42.

Peterson KA, Mole TB, Keong NCH, DeVito EE, Savulich G, Pickard JD, Sahakian BJ. Structural correlates of cognitive impairment in normal pressure hydrocephalus. Acta Neurol Scand. 2019 Mar;139(3):305-312.

Pesce A, Palmieri M, Scattolin A, Guerrini F, Czosnyka M, Czosnyka Z, Marano M, di Lazzaro V, Pompucci A, Iuliano L, Petrella G. Global Neurocognitive and Frontal Functions Analysis and Precision Intrathecal Pressure Measurement to Settle the Diagnostic Dilemma of the Normal Pressure Hydrocephalus: A Preliminary Experience. World Neurosurg. 2022 Nov;167:e1432-e1439.

Pfisterer WK, Aboul-Enein F, Gebhart E, Graf M, Aichholzer M, Mühlbauer M. Continuous intraventricular pressure monitoring for diagnosis of normal-pressure hydrocephalus. Acta Neurochir (Wien). 2007 Oct;149(10):983-90; discussion 990.

Pinto FC, Saad F, Oliveira MF, Pereira RM, Miranda FL, Tornai JB, Lopes MI, Ribas ES, Valinetti EA, Teixeira MJ. Role of endoscopic third ventriculostomy and ventriculoperitoneal shunt in idiopathic normal pressure hydrocephalus: preliminary results of a randomized clinical trial. Neurosurgery. 2013 May;72(5):845-53; discussion 853-4.

Poca MA, Mataró M, Del Mar Matarín M, Arikan F, Junqué C, Sahuquillo J. Is the placement of shunts in patients with idiopathic normal-pressure hydrocephalus worth the risk? Results of a study based on continuous monitoring of intracranial pressure. J Neurosurg. 2004 May;100(5):855-66.

Poca MA, Mataró M, Matarín M, Arikan F, Junqué C, Sahuquillo J. Good outcome in patients with normal-pressure hydrocephalus and factors indicating poor prognosis. J Neurosurg. 2005 Sep;103(3):455-63.

Poca MA, Solana E, Martínez-Ricarte FR, Romero M, Gándara D, Sahuquillo J. Idiopathic normal pressure hydrocephalus: results of a prospective cohort of 236 shunted patients. Acta Neurochir Suppl. 2012;114:247-53.

Pujari S, Kharkar S, Metellus P, Shuck J, Williams MA, Rigamonti D. Normal pressure hydrocephalus: long-term outcome after shunt surgery. J Neurol Neurosurg Psychiatry. 2008 Nov;79(11):1282-6.

Raftopoulos C, Deleval J, Chaskis C, Leonard A, Cantraine F, Desmyttere F, Clarysse S, Brotchi J. Cognitive recovery in idiopathic normal pressure hydrocephalus: a prospective study. Neurosurgery. 1994 Sep;35(3):397-404; discussion 404-5.

Raneri F, Zella MAS, Di Cristofori A, Zarino B, Pluderi M, Spagnoli D. Supplementary Tests in Idiopathic Normal Pressure Hydrocephalus: A Single-Center Experience with a Combined Lumbar Infusion Test and Tap Test. World Neurosurg. 2017 Apr;100:567-574.

Razay G, Wimmer M, Robertson I. Incidence, diagnostic criteria and outcome following ventriculoperitoneal shunting of idiopathic normal pressure hydrocephalus in a memory clinic population: a prospective observational cross-sectional and cohort study. BMJ Open. 2019 Dec 3;9(12):e028103.

Razay G, Vreugdenhil A, Liddell J. A prospective study of ventriculo-peritoneal shunting for idiopathic normal pressure hydrocephalus. J Clin Neurosci. 2009 Sep;16(9):1180-3.

Rydja J, Kollén L, Hellström P, Owen K, Lundgren Nilsson Å, Wikkelsö C, Tullberg M, Lundin F. Physical exercise and goal attainment after shunt surgery in idiopathic normal pressure hydrocephalus: a randomised clinical trial. Fluids Barriers CNS. 2021 Nov 22;18(1):51.

Rydja J, Eleftheriou A, Lundin F. Evaluating the cerebrospinal fluid tap test with the Hellström iNPH scale for patients with idiopathic normal pressure hydrocephalus. Fluids Barriers CNS. 2021 Apr 7;18(1):18.

Saadaldeen M, Jeppsson A, Hellström P, Blennow K, Zetterberg H, Wikkelsö C, Tullberg M. Idiopathic normal pressure hydrocephalus: associations between CSF biomarkers, clinical symptoms, and outcome after shunt surgery. Fluids Barriers CNS. 2025 May 19;22(1):51.

Saito M, Nishio Y, Kanno S, Uchiyama M, Hayashi A, Takagi M, Kikuchi H, Yamasaki H, Shimomura T, Iizuka O, Mori E. Cognitive profile of idiopathic normal pressure hydrocephalus. Dement Geriatr Cogn Dis Extra. 2011 Jan;1(1):202-11.

Saito A, Kamagata K, Ueda R, Nakazawa M, Andica C, Irie R, Nakajima M, Miyajima M, Hori M, Tanaka F, Arai H, Aoki S. Ventricular volumetry and free-water corrected diffusion tensor imaging of the anterior thalamic radiation in idiopathic normal pressure hydrocephalus. J Neuroradiol. 2020 Jun;47(4):312-317.

Sakurai A, Tsunemi T, Shimada T, Kawamura K, Nakajima M, Miyajima M, Hattori N. Effect of comorbid Parkinson's disease and Parkinson's disease dementia on the course of idiopathic normal pressure hydrocephalus. J Neurosurg. 2022 Mar 11;137(5):1302-1309.

Sand T, Bovim G, Grimse R, Myhr G, Helde G, Cappelen J. Idiopathic normal pressure hydrocephalus: the CSF tap-test may predict the clinical response to shunting. Acta Neurol Scand. 1994 May;89(5):311-6.

Savolainen S, Hurskainen H, Paljärvi L, Alafuzoff I, Vapalahti M. Five-year outcome of normal pressure hydrocephalus with or without a shunt: predictive value of the clinical signs, neuropsychological evaluation and infusion test. Acta Neurochir (Wien). 2002 Jun;144(6):515-23; discussion 523.

Shanks J, Markenroth Bloch K, Laurell K, Cesarini KG, Fahlström M, Larsson EM, Virhammar J. Aqueductal CSF Stroke Volume Is Increased in Patients with Idiopathic Normal Pressure Hydrocephalus and Decreases after Shunt Surgery. AJNR Am J Neuroradiol. 2019 Mar;40(3):453-459.

Shaw R, Everingham E, Mahant N, Jacobson E, Owler B. Clinical outcomes in the surgical treatment of idiopathic normal pressure hydrocephalus. J Clin Neurosci. 2016 Jul;29:81-6.

Shinoda N, Hirai O, Hori S, Mikami K, Bando T, Shimo D, Kuroyama T, Kuramoto Y, Matsumoto M, Ueno Y. Utility of MRI-based disproportionately enlarged subarachnoid space hydrocephalus scoring for predicting prognosis after surgery for idiopathic normal pressure hydrocephalus: clinical research. J Neurosurg. 2017 Dec;127(6):1436-1442.

Sindorio C, Abbritti RV, Raffa G, Priola SM, Germanò A, Visocchi M, Quattropani MC. Neuropsychological Assessment in the Differential Diagnosis of Idiopathic Normal Pressure Hydrocephalus. Acta Neurochir Suppl. 2017;124:283-288.

Sirkka J, Parviainen M, Jyrkkänen HK, Koivisto AM, Säisänen L, Rauramaa T, Leinonen V, Danner N. Upper limb dysfunction and activities in daily living in idiopathic normal pressure hydrocephalus. Acta Neurochir (Wien). 2021 Oct;163(10):2675-2683.

Skalický P, Mládek A, Vlasák A, Whitley H, Bradáč O. First experiences with Miethke M.blue® valve in iNPH patients. J Clin Neurosci. 2022 Apr;98:127-132.

Snöbohm C, Malmberg F, Freyhult E, Kultima K, Fällmar D, Virhammar J. White matter changes should not exclude patients with idiopathic normal pressure hydrocephalus from shunt surgery. Fluids Barriers CNS. 2022 May 23;19(1):35.

Solana E, Sahuquillo J, Junqué C, Quintana M, Poca MA. Cognitive disturbances and neuropsychological changes after surgical treatment in a cohort of 185 patients with idiopathic normal pressure hydrocephalus. Arch Clin Neuropsychol. 2012 May;27(3):304-17.

Sorteberg A, Eide PK, Fremming AD. A prospective study on the clinical effect of surgical treatment of normal pressure hydrocephalus: the value of hydrodynamic evaluation. Br J Neurosurg. 2004 Apr;18(2):149-57.

Spagnoli D, Innocenti L, Bello L, Pluderi M, Bacigaluppi S, Tomei G, Gaini SM. Impact of cerebrovascular disease on the surgical treatment of idiopathic normal pressure hydrocephalus. Neurosurgery. 2006 Sep;59(3):545-52; discussion 545-52.

Spanu G, Santagostino G, Marzatico F, Gaetani P, Silvani V, Rodriguez y Baena R. Idiopathic hydrocephalic dementia in aging brain the neurosurgical approach. Funct Neurol. 1989 Jul-Sep;4(3):293-8.

Spielmann H, Sippl C, Senger S, Oertel J. Predicting the long-term course: Shunt surgery results in idiopathic normal pressure hydrocephalus-a comprehensive study. Acta Neurochir (Wien). 2024 Oct 26;166(1):424.

St Louis PG, Boodoo S, Batz T, Clements-Lipofsky J. A retrospective outcomes review of patients with idiopathic normal pressure hydrocephalus treated with a low flow valve system. Interdisciplinary Neurosurgery. 2014 Oct 30;2(1):10–2.

Subramanian HE, Fadel SA, Matouk CC, Zohrabian VM, Mahajan A. The Utility of Imaging Parameters in Predicting Long-Term Clinical Improvement After Shunt Surgery in Patients with Idiopathic Normal Pressure Hydrocephalus. World Neurosurg. 2021 May;149:e1-e10.

Subramanian HE, Mahajan A, Sommaruga S, Falcone GJ, Kahle KT, Matouk CC. The Subjective Experience of Patients Undergoing Shunt Surgery for Idiopathic Normal Pressure Hydrocephalus. World Neurosurg. 2018 Nov;119:e46-e52.

Sundström N, Malm J, Laurell K, Lundin F, Kahlon B, Cesarini KG, Leijon G, Wikkelsø C. Incidence and outcome of surgery for adult hydrocephalus patients in Sweden. Br J Neurosurg. 2017 Feb;31(1):21-27.

Takeuchi T, Yajima K. Long-term 4 Years Follow-up Study of 482 Patients Who Underwent Shunting for Idiopathic Normal Pressure Hydrocephalus -Course of Symptoms and Shunt Efficacy Rates Compared by Age Group. Neurol Med Chir (Tokyo). 2019 Jul 15;59(7):281-286.

Thomas G, McGirt MJ, Woodworth G, Heidler J, Rigamonti D, Hillis AE, Williams MA. Baseline neuropsychological profile and cognitive response to cerebrospinal fluid shunting for idiopathic normal pressure hydrocephalus. Dement Geriatr Cogn Disord. 2005;20(2-3):163-8.

Thompson SD, Shand Smith JD, Khan AA, Luoma AMV, Toma AK, Watkins LD. Shunting of the over 80s in normal pressure hydrocephalus. Acta Neurochir (Wien). 2017 Jun;159(6):987-994.

Thomsen AM, Børgesen SE, Bruhn P, Gjerris F. Prognosis of dementia in normal-pressure hydrocephalus after a shunt operation. Ann Neurol. 1986 Sep;20(3):304-10.

Tisell M, Tullberg M, Hellström P, Blomsterwall E, Wikkelsø C. Neurological symptoms and signs in adult aqueductal stenosis. Acta Neurol Scand. 2003 May;107(5):311-7.

Tisell M, Tullberg M, Hellström P, Edsbagge M, Högfeldt M, Wikkelsö C. Shunt surgery in patients with hydrocephalus and white matter changes. J Neurosurg. 2011 May;114(5):1432-8.

Todisco M, Picascia M, Pisano P, Zangaglia R, Minafra B, Vitali P, Rognone E, Pichiecchio A, Ceravolo R, Vanacore N, Fasano A, Pacchetti C. Lumboperitoneal shunt in idiopathic normal pressure hydrocephalus: a prospective controlled study. J Neurol. 2020 Sep;267(9):2556-2566.

Tominaga H, Tokumoto H, Maeda S, Kawamura I, Sanada M, Kawazoe K, Taketomi E, Taniguchi N. High prevalence of lumbar spinal stenosis in cases of idiopathic normal-pressure hydrocephalus affects improvements in gait disturbance after shunt operation. World Neurosurg X. 2023 Jun 21;20:100236.

Trevisi G, Signorelli F, de Waure C, Stifano V, Sturdà C, Rapisarda A, Pompucci A, Mangiola A, Anile C. Intraventricular infusion test accuracy in predicting short- and long-term outcome of iNPH patients: a 10-year update of a three-decade experience at a single institution. Neurosurg Rev. 2021 Dec;44(6):3323-3334.

Tsakanikas D, Katzen H, Ravdin LD, Relkin NR. Upper extremity motor measures of Tap Test response in Normal Pressure Hydrocephalus. Clin Neurol Neurosurg. 2009 Nov;111(9):752-7.

Tseng PH, Huang LC, Huang XL, Huang BR, Lin SZ, Tsai ST, Huang HY. Blood-brain barrier-associated biomarker correlated with cerebral small vessel disease and shunt outcome in normal pressure hydrocephalus: a prospective cohort study. Int J Surg. 2024 Nov 1;110(11):6962-6971.

Valsecchi N, Mantovani P, Piserchia VA, Giannini G, Cevoli S, Aspide R, Oppi F, Milletti D, Cortelli P, Elder BD, Palandri G. The Role of Simultaneous Medical Conditions in Idiopathic Normal Pressure Hydrocephalus. World Neurosurg. 2022 Jan;157:e29-e39.

Virhammar J, Ahlgren A, Cesarini KG, Laurell K, Larsson EM. Cerebral Perfusion Does Not Increase after Shunt Surgery for Normal Pressure Hydrocephalus. J Neuroimaging. 2020 May;30(3):303-307.

Virhammar J, Laurell K, Cesarini KG, Larsson EM. The callosal angle measured on MRI as a predictor of outcome in idiopathic normal-pressure hydrocephalus. J Neurosurg. 2014 Jan;120(1):178-84.

Vivas-Buitrago T, Domingo R, Tripathi S, Herrera JP, Heemskerk J, Grewal S, Zalewski NL, Quinones-Hinojosa A, Reimer R, Wharen RE, Graff-Radford NR. In NPH, setting valve opening pressure close to lumbar puncture opening pressure decreases overdrainage. Neurol Neurochir Pol. 2020;54(6):531-537.

Vorstrup S, Christensen J, Gjerris F, Sørensen PS, Thomsen AM, Paulson OB. Cerebral blood flow in patients with normal-pressure hydrocephalus before and after shunting. J Neurosurg. 1987 Mar;66(3):379-87.

Wada T, Kazui H, Yamamoto D, Nomura K, Sugiyama H, Shimizu Y, Yoshida T, Yoshiyama K, Yamashita F, Kishima H, Yoshimine T, Takeda M. Reversibility of brain morphology after shunt operations and preoperative clinical symptoms in patients with idiopathic normal pressure hydrocephalus. Psychogeriatrics. 2013 Mar;13(1):41-8.

Wesner E, Etzkorn L, Bakre S, Chen J, Davis A, Zhang Y, Yasar S, Rao A, Luciano M, Wang J, Moghekar A. The Clinical Utility of the MOCA in iNPH Assessment. Front Neurol. 2022 May 23;13:887669.

Wetzel C, Goertz L, Schulte AP, Goldbrunner R, Krischek B. Minimizing overdrainage with flow-regulated valves - Initial results of a prospective study on idiopathic normal pressure hydrocephalus. Clin Neurol Neurosurg. 2018 Oct;173:31-37.

Wikkelsø C, Hellström P, Klinge PM, Tans JT; European iNPH Multicentre Study Group. The European iNPH Multicentre Study on the predictive values of resistance to CSF outflow and the CSF Tap Test in patients with idiopathic normal pressure hydrocephalus. J Neurol Neurosurg Psychiatry. 2013 May;84(5):562-8.

Wikkelsø C, Andersson H, Blomstrand C, Lindqvist G, Svendsen P. Normal pressure hydrocephalus. Predictive value of the cerebrospinal fluid tap-test. Acta Neurol Scand. 1986 Jun;73(6):566-73.

Williams MA, Nagel SJ, Golomb J, Jensen H, Dasher NA, Holubkov R, Edwards RJ, Luciano MG, Zwimpfer TJ, Katzen H, Moghekar A, Wisoff JH, McKhann GM, Hamilton MG. Safety and effectiveness of the assessment and treatment of idiopathic normal pressure hydrocephalus in the Adult Hydrocephalus Clinical Research Network. J Neurosurg. 2022 Mar 11;137(5):1289-1301.

Wolfsegger T, Hauser A, Wimmer S, Neuwirth K, Assar H, Topakian R. A comprehensive clinico-radiological, neuropsychological and biomechanical analysis approach to patients with idiopathic normal pressure hydrocephalus. Clin Neurol Neurosurg. 2021 Feb;201:106402.

Wu D, Moghekar A, Shi W, Blitz AM, Mori S. Systematic volumetric analysis predicts response to CSF drainage and outcome to shunt surgery in idiopathic normal pressure hydrocephalus. Eur Radiol. 2021 Jul;31(7):4972-4980.

Yamada SM, Masahira N, Kawanishi Y, Fujimoto Y, Shimizu K. Preoperative acetazolamide SPECT is useful for predicting outcome of shunt operation in idiopathic normal pressure hydrocephalus patients. Clin Nucl Med. 2013 Sep;38(9):671-6.

Yamada S, Kimura T, Jingami N, Atsuchi M, Hirai O, Tokuda T, Miyajima M, Kazui H, Mori E, Ishikawa M; SINPHONI-2 Investigators. Disability risk or unimproved symptoms following shunt surgery in patients with idiopathic normal-pressure hydrocephalus: post hoc analysis of SINPHONI-2. J Neurosurg. 2017 Jun;126(6):2002-2009.

Yamamoto D, Kazui H, Wada T, Nomura K, Sugiyama H, Shimizu Y, Yoshiyama K, Yoshida T, Kishima H, Yamashita F, Yoshimine T, Takeda M. Association between milder brain deformation before a shunt operation and improvement in cognition and gait in idiopathic normal pressure hydrocephalus. Dement Geriatr Cogn Disord. 2013;35(3-4):197-207.

Yang L, Wang X, Li Y, Chen L, Yan Z, She L, Dong J. The Clinical Effect of Postoperative Hyperbaric Oxygen Therapy on Idiopathic Normal Pressure Hydrocephalus: A Retrospective and Comparative Analysis of 61 Patients with Ventriculoperitoneal Shunt. World Neurosurg. 2017 Aug;104:376-380.

Yang F, Hickman TT, Tinl M, Iracheta C, Chen G, Flynn P, Shuman ME, Johnson TA, Rice RR, Rice IM, Wiemann R, Johnson MD. Quantitative evaluation of changes in gait after extended cerebrospinal fluid drainage for normal pressure hydrocephalus. J Clin Neurosci. 2016 Jun;28:31-7.

Yang F, Yang L, Fang X, Deng Y, Mao R, Yan A, Wei W. Increased Cerebrospinal Fluid Levels of Soluble Triggering Receptor Expressed on Myeloid Cells 2 and Chitinase-3-Like Protein 1 in Idiopathic Normal-Pressure Hydrocephalus. J Alzheimers Dis. 2023;93(4):1341-1354.

Yang Y, Yan M, Liu X, Li S, Lin G. Alterations of Glymphatic System Before and After Shunt Surgery in Patients With Idiopathic Normal Pressure Hydrocephalus: A Longitudinal Study. Eur J Neurol. 2025 May;32(5):e70200.

Yasar S, Jusue-Torres I, Lu J, Robison J, Patel MA, Crain B, Carson KA, Hoffberger J, Batra S, Sankey E, Moghekar A, Rigamonti D. Alzheimer's disease pathology and shunt surgery outcome in normal pressure hydrocephalus. PLoS One. 2017 Aug 7;12(8):e0182288.

Yerneni K, Karras CL, Larkin CJ, Weiss H, Hopkins B, Kesavabhotla K, Potts MB, Tate MC, Bloch O. Lumboperitoneal shunts for the treatment of idiopathic normal pressure hydrocephalus. J Clin Neurosci. 2021 Apr;86:1-5.
